# Inferring oscillatory modulation in neural spike trains

**DOI:** 10.1371/journal.pcbi.1005596

**Published:** 2017-10-06

**Authors:** Kensuke Arai, Robert E. Kass

**Affiliations:** 1 Department of Statistics, Carnegie Mellon University, Pittsburgh, Pennsylvania, United States of America; 2 Center for the Neural Basis of Cognition, Pittsburgh, Pennsylvania, United States of America; 3 Machine Learning Department, Carnegie Mellon University, Pittsburgh, Pennsylvania, United States of America; University of Tübingen and Max Planck Institute for Biologial Cybernetics, GERMANY

## Abstract

Oscillations are observed at various frequency bands in continuous-valued neural recordings like the electroencephalogram (EEG) and local field potential (LFP) in bulk brain matter, and analysis of spike-field coherence reveals that spiking of single neurons often occurs at certain phases of the global oscillation. Oscillatory modulation has been examined in relation to continuous-valued oscillatory signals, and independently from the spike train alone, but behavior or stimulus triggered firing-rate modulation, spiking sparseness, presence of slow modulation not locked to stimuli and irregular oscillations with large variability in oscillatory periods, present challenges to searching for temporal structures present in the spike train. In order to study oscillatory modulation in real data collected under a variety of experimental conditions, we describe a flexible point-process framework we call the Latent Oscillatory Spike Train (LOST) model to decompose the instantaneous firing rate in biologically and behaviorally relevant factors: spiking refractoriness, event-locked firing rate non-stationarity, and trial-to-trial variability accounted for by baseline offset and a stochastic oscillatory modulation. We also extend the LOST model to accommodate changes in the modulatory structure over the duration of the experiment, and thereby discover trial-to-trial variability in the spike-field coherence of a rat primary motor cortical neuron to the LFP theta rhythm. Because LOST incorporates a latent stochastic auto-regressive term, LOST is able to detect oscillations when the firing rate is low, the modulation is weak, and when the modulating oscillation has a broad spectral peak.

## Author summary

Oscillatory modulation of neural activity in the brain is widely observed under conditions associated with a variety of cognitive tasks and mental states. Within individual neurons, oscillations may be uncovered in the moment-to-moment variation in neural firing rate. This, however, is often challenging because many factors may affect fluctuations in neural firing rate and, in addition, neurons fire irregular sets of action potentials, or spike trains, due to an unknown combination of meaningful signals and extraneous noise. We have devised a statistical Latent Oscillatory Spike Train (LOST) model with accompanying model-fitting technology, that is able to detect subtle oscillations in spike trains by taking into account both spiking noise and temporal variation in the oscillation itself. The method couples two techniques developed for other purposes in the literature on Bayesian analysis. Using data simulated from theoretical neurons and real data recorded from cortical motor neurons, we demonstrate the method’s ability to track changes in the modulatory structure of the oscillation across experimental trials.

## Introduction

Neural oscillations have generated considerable interest for their roles in cognition and as indicators for disease [1–14]. Electroencephalograms (EEGs) and local field potential (LFPs) recordings have revealed transient oscillations in many cortical and subcortical structures, to which neurons both near and far from the LFP recording site show phase preferences in spiking. Groups of neurons that are locked to a common oscillation, and are therefore active in tightly confined temporal windows, may define a cell assembly whose synchronous spiking could select and activate afferent structures [15–17]. Such transient cell assemblies may form and disband as objects are attended to in the visual scene [3, 6–8, 11], as preparation for movements are made [2, 13], or during choice points for reward [9]. Prominent slow oscillations are observed as patients undergo anesthesia [10], and exhibit changes in dynamics during transitions in unconscious brain state [14]. Furthermore, strong oscillatory signals are prominent in neurological disorders such as Parkinson’s disease, and can be used to characterize pathophysiology and its relation to behavior [12, 18, 19]. The oscillatory structure of individual neurons can thus provide insight into the dynamics of cognitive processing and motor planning, and their implementation in health and disease. However, identifying oscillations in neural spike trains, particularly within individual experimental trials, can be challenging because they occur in conjunction with both non-oscillatory fluctuations in neural firing rate and extraneous noise. To tease apart multiple factors that affect spiking activity, statisticians have suggested the use of point process models together with modern regression methods associated with generalized linear model (GLM) technology [20, 21]. In this paper we develop a method that can find oscillations in spike trains even when the signal is comparatively weak, and can track changes in oscillatory behavior across trials.

The starting point for our approach is the observation by Smith and Brown [22] that, for many purposes, evolving neural firing rates can be described using state-space models imbedded in point processes. We modify the models used by Smith and Brown, replacing their first-order autoregressive processes with higher-order processes [23] that can fit oscillations found in spiking neurons. This requires careful attention to the form of the higher-order autoregressive process. We take advantage of a Bayesian time-series decomposition introduced by Huerta and West [24], using prior probability distributions to constrain the fit so that it has appropriate power spectral content. We also use a Gibbs sampling method developed recently in a different context [25] to compute posterior distributions efficiently.

Our approach is superficially related to that of Allcroft *et al.* [26], who used autoregressive moving average models to estimate spectral content from censored data, but their method does not accommodate easily the special situation we face with spiking neurons, including history effects that can account for hard and soft refractory periods. Point process models employing the GLM framework [21, 27] have been employed to explain the spiking behavior by taking into account spiking refractoriness, behavioral and stimulus-induced non-stationarities, and spiking of neighboring neurons [20]. A series of investigations [12, 18, 19] have added long-term history effects to capture oscillation in the history dependence and have also used a state-space smoothing algorithm to track changes in the oscillatory dynamics over time. Eden et al used a long history to capture both, but as we shall see, LOST can better capture the irregularities present in realistic biological oscillations. Our approach is based on what we call the Latent Oscillatory Spike Train (LOST) model, which not only includes terms to describe the spiking refractoriness, event (behavior or stimulus)-locked effects, and trial-to-trial variability in firing rate, but also explicitly models the dynamics of the modulating oscillation itself. However, the latent state cannot capture the discontinuous change in the spiking probability that occurs after every spike, which the aforementioned GLM models do well in characterizing, so we utilize a short-term spiking history to capture the refractory period, while also introducing the latent state to model the oscillatory dynamics.

Oscillatory structure is often considerably degraded when it is observed as a spike train. On the one hand, if the firing rate is low, or the oscillatory modulation is weak, noise associated with erratic spiking will dominate. On the other hand, even if the modulation is strong and the firing rate is high, the oscillatory signal may itself be unsteady, with substantial variation in period from cycle to cycle. LOST accommodates both spiking noise and oscillatory variation by imbedding into the point process intensity function a latent auto-regressive process, and then imposing a suitable soft constraint (in the form of a prior probability distribution) on the oscillatory dynamics. We provide details of the model, along with the posterior sampling scheme and a discussion on how to interpret the posterior samples, and study its effectiveness with leaky integrate-and-fire neural simulations. We then demonstrate the ability of the LOST framework to find interesting trial-to-trial variation in motor cortical neuron during a lever-pulling task.

## Model

We seek to extract the underlying oscillation from the binary (*M* × *N*) matrix **y** representing the binned spiking data from a single neuron during *M* repetitions of identical behavior or stimulus presentations of time duration *N*. Because the spiking times are different for each trial, even though the behavior or stimulus are identical, we construct a probabilistic model of the spiking.

A point process models the probability of observing a spike at time *t*. The conditional intensity function (CIF) completely specifies the point process, and is defined as

$$\begin{array}{c} \lambda \left[t|\Theta ,H\left(t\right)\right]=\lim_{\Delta t\to 0}\frac{P[N(t+\Delta t)-N(t)=1|\Theta ,H(t)]}{\Delta t}, \end{array}$$ (1)

where *P*[⋅|⋅] is a conditional probability conditioned on the past spiking history *H*(*t*) and model parameters Θ [21], making the point process CIF a spike history-dependent generalization of a general inhomogeneous Poisson process. We discretize the problem, and note that λ[*t*|*H*(*t*)]Δ*t* is the approximate binary probability of observing a single spike in a small time interval Δ*t*. In the discrete time representation, the likelihood of the spike train is then expressed as a product of conditionally independent Bernoulli events

$$P[y|\Theta ,H(t)]=\prod_{mn}^{MN}p{\left[y_{mn}=1|\Theta ,H(t)\right]}^{y_{mn}}p{\left[y_{mn}=0|\Theta ,H(t)\right]}^{1-y_{mn}}$$ (2)

The binary spiking probability is expressed as a sigmoidal function of a sum of several predictors, including a history term **λ**^*R*^, modeling the refractoriness following a spike. In the simplest case, the predictors will be the shared trial-average effect (TAE) **f** = ***β***^*T*^***α***, with ***β*** being the (*K*_*β*_ × *N*) matrix of the *K*_*β*_ B-spline basis vectors and ***α*** the *K*_*α*_ weights, the history term **λ**^*R*^ = **H**^*T*^ **v**, with **H** being the (*K*_*H*_ × *N*) matrix of the *K*_*H*_ B-spline basis vectors and **v** the *K*_*H*_ weights, the *M*-dimensional trial-specific baseline offset vector ***μ***, and a time-dependent latent oscillational modulation **x**. The TAE is closely related to the peristimulus time histogram (PSTH) often employed in neuroscience. Together, the binary spiking probability for trial *m* at time *n* is

$$\begin{array}{cc} p(y_{mn}|x_{mn},H_{n,l},\Theta ) & =\frac{{\left(e^{x_{mn}+\mu_{m}+f_{n}+\lambda_{l\left(m,n\right)}^{R}}\right)}^{y_{mn}}}{1+e^{x_{mn}+\mu_{m}+f_{n}+\lambda_{l\left(m,n\right)}^{R}}}, \end{array}$$ (3a)

$$\begin{array}{cc} x_{mn} & =\sum_{j=1}^{p}F_{j}x_{m,n-j}+\epsilon_{mn}, \end{array}$$ (3b)

where $l\left(m,n\right)\equiv n-L\left(m,n\right)\in Z_{\geq 0}$, where *L*(*m*, *n*) is the last spike time before time *n* of the *m*th trial. From the data, we infer the parameters and the latent oscillation **x**, which is an AR(*p*) process, the *F*_1:*p*_ the *p* AR coefficients and *ϵ*_*mn*_ a Gaussian random variable ∼ *N*(0, *σ*^2^), where *σ*^2^ is the innovation variance. In what follows, we will often refer to the latent oscillation as simply oscillation. We also note that the inferred **x** may be spectrally broad, or have a very small amplitude, and may not be represent a signal that would commonly be called an oscillation.

The simplest oscillating AR model is of order 2 with complex eigenvalues, and may be a good model for a low dimensional oscillatory dynamical system. However fitting a model to noisy observed data utilizes noisy lagged values. The implied model for such a time series is an ARMA process, and an AR(2) would fail to capture all the structure in the observed data [24], so we approximate the ARMA process with a higher-order AR(p) process.

The spectra of AR(*p*) models is closely related to its *p* = 2*C* + *R* roots of the characteristic polynomial of *F*_1:*p*_, or equivalently to the eigenvalues of the matrix **F** in Eq 6. The time-series decomposition [24] approach restricts the AR(*p*) to those with *C* imaginary AR(2) components and *R* real AR(1) components, ie only models that can accommodate the spectral features we believe are present in the data. We work through the rest of the paper using a generic structure that has proven to be a generally reasonable component structure (*C* = 4 and *R* = 1) for a wide range of simulation parameters and for real data, though model order and component structure can also be inferred from the data.

### Model fitting

Gibbs sampling [28] and data augmentation [29, 30] are used to infer the model parameters Θ = [*F*_1:*p*_, *σ*^2^, ***α***, ***μ***, **v**] and the oscillation **x**. The spike history and TAE are functions of time relative to the last spike of an event or behavior, respectively, and they are estimated by parameterizing a subset of continuous functions with splines. The estimation requires us to choose a set of basis splines, which we choose heuristically, using some prior belief about their general shape to choose the knot locations. In the current work, the number and locations of the knots are not found with the Gibbs sampling procedure, so we briefly describe how they are determined before we describe the Gibbs sampling procedure.

#### Short-term spike history λ^*R*^

Neurons typically have a refractory period following an action potential, when sodium channels in the membrane become inactive and spiking is strongly suppressed. As the channels reactivate, the neuron may experience a period of rebound excitation, where spiking probability becomes enhanced, before returning to some baseline level. This effect can be modeled by a modulation of probability since the last spike. We employ cubic splines to express the refractory modulation function, with knots placed where we would like to constrain function values, where we believe the function reaches extremum values, and places in between. The interspike interval (ISI) histogram reflects combined effects of both the spiking history and oscillation, but at the smaller intervals, the refractory inhibition and rebound excitation are rapidly changing, possibly from 0 to well over 1, while a biologically plausible oscillation varies over a more limited range of values centered around 1. The shape of the ISI histogram for smaller intervals should reflect the refractory modulation function more than the oscillation, so we place history spline knots based on features of the ISI near 0. In the case the ISI histogram has a minimum at 0 followed by a maxima, we place knots at *t* = Δ, one knot at the first maxima, one knot at the mean of the ISIs, and knots at the 70th and 80th percentiles of the ISI distribution. We further have knots at the 97th and at 100ms set to 0, constraining the asymptotic value of the history function. Further, if the bins of the ISI at time Δ are empty, we set *v*_0_ = −6 to prevent it from taking unconstrained negative values. If the histogram at 0 is the maximum value, we leave *v*_0_ unconstrained.

#### TAE spline basis *β*

To represent a smooth, arbitrary-shaped curve, we use the piecewise cubic B-spline as basis functions. Choosing the appropriate number and locations of basis functions is vital to represent the curve well, and too many points will result in overfitting. We determine the number and locations prior to the Gibbs procedure, by assuming that the shape of the TAE **f** in Eq (3a) is similar to the empirical PSTH. We randomly vary the number and locations of the spline knots *β* and find a set which minimizes the squared error with the PSTH under the constraint that no more than 9 knots are to be used for the data presented in this paper. The shape of the PSTH can be used as a guide to guess the rough number of knots necessary, and the constraint can be changed accordingly for the spike train under investigation.

#### Posterior simulation

We now introduce the posterior distribution, from which the conditional posteriors necessary for Gibbs sampling, and the generating distributions necessary for data augmentation, are derived. Our model is a state-space model with a point-process observable, and a latent oscillation evolving as an AR(*p*)-process. The posterior distribution of the parameters Θ is

p(Θ|y)∝p(Θ)∫p(y,x|Θ)dx=p(Θ)∫p(y|x,Θ)p(x|Θ)dx (4)

For the AR(*p*) model, *p*(**x**|Θ) does not have a simple expression. However, if **x** were a Markov process, then *p*(**x**|Θ) could be expressed recursively in terms of the initial probability at time 0. AR(*p*) can be made a Markov process if we define a new (*N* × *M* × *p*) matrix **X**, where **X** *_mn_* = (*x*_*m*,*n*−1_, …, *x* *_m,n−p_*)^*T*^. Eq (3b) is then

$${\text{X}}_{mn}=F{\text{X}}_{m,n-1}+\epsilon_{mn},$$ (5)

where

$$\begin{array}{cc} \text{F} & =(\begin{array}{ccccc} F_{1} & F_{2} & \ldots & F_{p-1} & F_{p}\\ 1 & 0 & \ldots & 0 & 0\\ \vdots & \vdots & \vdots & \vdots & \vdots \\ 0 & 0 & \ldots & 1 & 0 \end{array}),\;\text{and}\;\;\epsilon_{mn}=(\begin{array}{c} \epsilon_{mn}\\ 0\\ \vdots \\ 0 \end{array}). \end{array}$$ (6)

(*N* × *M*) matrix **x** is the oscillation we would like to infer, and likely corresponds to oscillations of the membrane potential due to oscillating synaptic inputs [31, 32]. Eq (4) then becomes

$$p(\Theta |y)\propto p\left(\Theta \right)\int_{\text{X}}\overset{M}{\underset{m=1}{\prod }}[\overset{N}{\underset{n=0}{\prod }}p(y_{mn}|x_{mn},H_{n,l},\Theta )\overset{N}{\underset{n^{\prime }=1}{\prod }}p({\text{X}}_{mn^{\prime }}|{\text{X}}_{m,n^{\prime }-1},\Theta )p({\text{X}}_{m0}|\Theta )d\text{X}].$$ (7)

### Conditional posteriors and data augmentation for Gibbs sampling

We jointly sample model parameters and latent states from the joint posterior distribution, Eq (4), using Gibbs sampling with data augmentation. The conditional posterior distribution can be read off from the full joint posterior distribution by considering all parameters fixed, except the parameter whose conditional posterior we are interested in. Our problem deviates from the standard Gibbs sampling by the missing data **x** in Eq (4), and we utilize the data augmentation strategy [29, 30], a scheme of simplifying analysis by augmenting the observed data with missing values, to generate samples of **X** from the predictive distribution *p*(**X**|**y**, Θ) in between sampling parameters from the standard conditional posteriors. Even with samples of **X** in hand, the posterior distribution does not yield conditional posteriors that can be sampled easily. The recently developed Pólya-Gamma data augmentation scheme [25] allows sampling from simple Gaussian conditional posteriors at the cost of introducing the *M* × *N* matrix of Pólya-Gamma variables ***ω*** through the following identity for the spike probability:

$$\begin{array}{cc} & p\left(y_{mn}|\Theta ,H_{n,l},x_{mn}\right)=\frac{{\left(e^{x_{mn}+\mu_{m}+\lambda_{l(m,n)}^{R}+f_{n}}\right)}^{y_{mn}}}{1+e^{x_{mn}+\mu_{m}+\lambda_{l(m,n)}^{R}+f_{n}}}=\frac{1}{2}e^{\kappa_{mn}\left(x_{mn}+f_{n}+\mu_{m}+\lambda_{l(m,n)}^{R}\right)}\times \\ & \int_{0}^{\infty }e^{-\omega_{mn}\frac{{\left(x_{mn}+f_{n}+\mu_{m}+\lambda_{l(m,n)}^{R}\right)}^{2}}{2}}PG\left(\omega_{mn}|b=1,z=0\right)d\omega_{mn}\\ & \propto \int_{0}^{\infty }N(x_{mn}+f_{n}+\mu_{m}+\lambda_{l(m,n)}^{R}|\frac{\kappa_{mn}}{\omega_{mn}},\frac{1}{\omega_{mn}})PG\left(\omega_{mn}|b=1,z=0\right)d\omega_{mn} \end{array}$$ (8)

where *PG*(*ω*_*mn*_|*b* = 1, *z* = 0) is the Pólya-Gamma distribution with parameters *b* = 1 and *z* = 0, and $\kappa \equiv \text{y}-\frac{1}{2}$.

With the introduction of the Pólya-Gamma variables, the joint posterior Eq (4) becomes

$$\begin{array}{l} p(\Theta |y)\propto p\left(\Theta \right)\int_{\text{X}}[\overset{M}{\underset{m=1}{\prod }}\overset{N}{\underset{n^{\prime }=1}{\prod }}p({\text{X}}_{mn^{\prime }}|{\text{X}}_{m,n^{\prime }-1},\Theta )p({\text{X}}_{m0}|\Theta )\times \\ \prod_{n=0}^{N}\int_{0}^{\infty }N(x_{mn}+f_{n}+\mu_{m}+\lambda_{l\left(m,n\right)}^{R}|\frac{\kappa_{mn}}{\omega_{mn}},\frac{1}{\omega_{mn}})PG(\omega_{mn}|b=1,z=0)d\omega_{mn}]d\text{X}, \end{array}$$ (9)

from which we derive the conditional posteriors for the parameters and the predictive distributions for the augmented variables. We refer to the set of augmented variables as **V**^+^ = {**X**, **ω**}, and denote by Θ_\*x*_ to be the set of all parameters except parameter *x*.

#### Sampling *μ*

The conditional posterior of ***μ*** ∼ *N* (**M_*μ*_**, **Σ_*μ*_**) is a multivariate Gaussian, and if we constrain ***μ*** such that $\sum_{m}^{M}\mu_{m}=0$, and set $\mu_{1}=-\sum_{m=2}^{M}\mu_{m}$, it becomes

$$\begin{array}{l} p(\mu |\Theta_{\backslash \mu },V^{+})\propto \text{exp}\{\sum_{m>1,n}^{M,N}-\frac{\omega_{mn}}{2}{\left[\left(x_{mn}+\mu_{m}+f_{n}+\lambda_{l\left(m,n\right)}^{R}\right)-\frac{\kappa_{mn}}{\omega_{mn}}\right]}^{2}\\ -\sum_{n}^{N}\frac{\omega_{1n}}{2}{\left[\left(x_{1n}-\sum_{m^{\prime }>1}^{M}\mu_{m^{\prime }}+f_{n}+O_{1n}\right)-\frac{\kappa_{1n}}{\omega_{1n}}\right]}^{2}-\frac{1}{2}{\left(\mu -\mu_{\mu }\right)}^{T}D_{\mu }^{-1}\left(\mu -\mu_{\mu }\right)\}\\ =\text{exp}\left(-\frac{1}{2}Q_{\mu }\right), \end{array}$$ (10)

where $O_{mn}\equiv \frac{\kappa_{mn}}{\omega_{mn}}-x_{mn}-f_{n}-\lambda_{l(m,n)}^{R}$. The covariance can be read off from the quadratic term,

$$\begin{array}{c} \Sigma_{\mu }^{-1}=(\begin{array}{cccc} \sum_{n}\left(\omega_{1n}+\omega_{2n}\right) & \sum_{n}\omega_{1n} & ... & \sum_{n}\omega_{1n}\\ \sum \omega_{1n} & \sum_{n}\left(\omega_{1n}+\omega_{3n}\right) & ... & \sum_{n}\omega_{1n}\\ \vdots & \vdots & \vdots & \vdots \\ \sum_{n}\omega_{1n} & \sum_{n}\omega_{1n} & ... & \sum_{n}\left(\omega_{1n}+\omega_{Mn}\right) \end{array}) \end{array}$$ (11)

The mean **M_*μ*_** is at the minimum of *Q***_*μ*_**, which can be found by setting $\frac{\partial Q_{\mu }}{\partial \mu }=0$, or $\Sigma_{\mu }^{-1}M_{\mu }=g$, where *g*_*i*_ = ∑_*n*_ *ω*_1*n*_*O*_1*n*_ − *ω*_*in*_*O*_*in*_.

#### Sampling v

**v** are the vector spline weights for the spike history, and {*I*_*H*_} = {1, …, *K*_*H*_} its component indices. We impose our prior belief about the timescale and shape of the spike history term by keeping some of the knots fixed, while re-weighting the remaining ones. The indices of the fixed ones we denote {*I*_*f*_} and the adjustable one {*I*_*a*_}, with {*I*_*f*_} ∪ {*I*_*a*_} = {*I*_*H*_} and {*I*_*f*_} ∩ {*I*_*a*_} = ∅. We use the shorthand notations **a** to be the vector comprised of components {*I*_*a*_} of **v**, and {*I*_*a*_} = {*a*(1), *a*(2), …*a*(|{*I*_*a*_}|)}, its numerical values. Then the conditional posterior of **a** ∼ *N* (**M**_**a**_, **Σ**_**a**_) is a multivariate Gaussian. Defining $O_{mn}\equiv (\frac{\kappa_{mn}}{\omega_{mn}}-x_{mn}-f_{n}-\mu_{m})$, it is

$$\begin{array}{ll} p(a|\Theta_{\backslash v},V^{+}) & \propto \text{exp}\left[-\sum_{m}^{M}\left\{\sum_{n}^{N}\frac{\omega_{mn}}{2}{\left(\lambda_{l\left(m,n\right)}^{R}-O_{mn}\right)}^{2}+\ldots \right\}-\frac{1}{2}{\left(v-\mu_{v}\right)}^{T}D^{-1}\left(v-\mu_{v}\right)\right]\\ & =\sum_{m}^{M}\left\{\sum_{n}^{N}-\frac{\omega_{mn}}{2}{\left(\sum_{j}^{K_{H}}H_{l\left(m,n\right),j}v_{j}-O_{mn}\right)}^{2}\right\}-\frac{1}{2}\sum_{j}\frac{v_{j}^{2}-2v_{j}\mu_{v_{j}}+\mu_{v_{j}}}{\sigma_{v_{j}}^{2}}\\ & =\text{exp}\left(-\frac{1}{2}Q_{a}\right). \end{array}$$ (12)

The mean **M_a_** of the adjustable knots is the solution of the set of linear equations whose *i*th equation, *i* ∈ {*I*_*a*_} is

$$\begin{array}{cc} & \sum_{j\in \{I_{a}\}}2(\sum_{m}^{M}\sum_{n}^{N}\omega_{mn}H_{l(m,n),j}H_{l(m,n),i}+\frac{\delta_{ji}}{\sigma_{v_{j}}^{2}})v_{j}=\\ & =2(\sum_{m}^{M}\sum_{n}^{N}\omega_{mn}H_{l(m,n),i}+\frac{\mu_{v_{i}}}{\sigma_{v_{i}}^{2}})-2\sum_{j\in \{I_{f}\}}(\sum_{m}^{M}\sum_{n}^{N}\omega_{mn}H_{l(m,n),j}H_{l(m,n),i}+\frac{\delta_{ji}}{\sigma_{v_{j}}^{2}})v_{j} \end{array}$$

The covariance can be read off from the quadratic term, and is

$$\begin{array}{c} \Sigma_{a}^{-1}=\left(\begin{array}{ccc} \underset{mn}{\sum }\omega_{mn}H_{l\left(m,n\right),a\left(1\right)}^{2}+\frac{1}{\sigma_{v_{a\left(1\right)}}^{2}} & \underset{mn}{\sum }\omega_{mn}H_{l\left(m,n\right),a\left(1\right)}H_{l\left(m,n\right),a\left(2\right)} & \ldots \\ \underset{mn}{\sum }\omega_{mn}H_{l\left(m,n\right),a\left(1\right)}H_{l\left(m,n\right),a\left(2\right)} & \underset{mn}{\sum }\omega_{mn}H_{l\left(m,n\right),a\left(2\right)}^{2}+\frac{1}{\sigma_{v_{a\left(2\right)}}^{2}} & \ldots \\ \vdots & \vdots & \ddots \end{array}\right) \end{array}$$ (13)

#### Sampling *α*

The conditional posterior of ***α*** ∼ *N* (**M**_***α***_, **Σ**_***α***_) is multivariate Gaussian,

$$p(\alpha |\Theta_{\backslash \alpha },V^{+})\propto \text{exp}\{\overset{M,N}{\underset{m\neq 1,n}{\sum }}-\frac{\omega_{mn}}{2}{\left[\left(x_{mn}+\mu_{m}+f_{n}+\lambda_{l\left(m,n\right)}^{R}\right)-\frac{\kappa_{mn}}{\omega_{mn}}\right]}^{2}$$ (14)

By rearranging, we can easily calculate the mean and variance of the conditionals for ***α***. If *n* or *m* is kept fixed in the sum, this is a quadratic form of *M* or *N*-dim vectors with a diagonal covariance matrix, and ***α*** ∼ *N* (**M**, **Σ**), For ***α***,

$$\begin{array}{ll} M_{\alpha } & =\mu_{\alpha }+D_{\alpha }\beta {\left(\beta^{T}D_{\alpha }\beta +W\right)}^{-1}\left(O-\beta^{T}\mu_{\alpha }\right)\\ \sum {}_{\alpha }^{-1} & =\beta W^{-1}\beta^{T}+D_{\alpha }^{-1}\\ W^{-1} & =\sum_{m}W_{m}^{-1},\;\text{where}\;{\left(W_{m}^{-1}\right)}_{nn}=\omega_{mn}\\ O & =W\sum_{m}W_{m}^{-1}O_{m},\;\text{where}\;{\left(O_{m}\right)}_{n}=\left(\frac{\kappa_{mn}}{\omega_{mn}}-x_{mn}-\lambda_{l\left(m,n\right)}^{R}-\mu_{m}\right). \end{array}$$ (15)

***μ*_*α*_** and **D*_α_*** are prior mean and covariances for ***α***. Aside from the smoothness imposed by the restriction to the number of knots, we have no prior knowledge about the offsets and knot weights, so we set the means to 0 and the covariances to be broad.

#### Sampling *σ*^2^

The conditional posterior distribution of the innovation variance is

$$\begin{array}{c} p(\sigma^{2}|\Theta_{\backslash \sigma^{2},X,\omega })\propto {\left(\frac{1}{\sqrt{2\pi \sigma^{2}}}\right)}^{M\left(N-1\right)}\text{exp}\left(-\frac{1}{2\sigma^{2}}\sum_{mn}{\left[x_{mn}-{\left(FX_{m,n-1}\right)}_{1}\right]}^{2}\right), \end{array}$$ (16)

and is in the form of an inverse Gamma distribution. We choose a conjugate inverse Gaussian prior with parameters *α*_*σ*^2^_ and *β*_*σ*^2^_, and sample as

$$\begin{array}{c} \sigma^{2}∼IG[\sigma^{2}|\alpha_{\sigma^{2}}+\frac{M\left(N-1\right)+2}{2},\beta_{\sigma^{2}}+\frac{1}{2}\sum_{mn}{\left[x_{mn}-{\left(FX_{m,n-1}\right)}_{1}\right]}^{2}] \end{array}$$ (17)

As with the offset and spline weights, we have no *apriori* knowledge about an appropriate range of values for *σ*^2^. In order to minimize the effect of the prior, we choose a small values for *α*_*σ*^2^_ (relative to the amount of data), and *β*_*σ*^2^_, so that small values of *σ*^2^ can also be sampled.

#### Sampling *F*_1:*p*_

Without further assumptions on the latent dynamics [33], the conditional posterior is a simple multivariate normal distribution. However, for application to spiking data, this naive approach often does not produce desired results for two main reasons. First, the constrained sampling of the coefficients to the stationary regime is inefficient, due to the nontrivial shape of the surface of the stationary manifold [24]. Second, our observations are noisy and sparse, and maximum likelihood using a flat prior may not find a state with an oscillatory character. The component decomposition [24] approach addresses both of these concerns, and allows us to specifically search for models with an oscillatory character through the imposition of an informative prior.

We seek to sample

$$\begin{array}{c} p(F_{1:p}|\Theta_{\backslash F_{1:p}},V^{+})\propto \text{exp}\left(-\frac{1}{2\sigma^{2}}\sum_{mn}{\left[x_{mn}-{\left(FX_{m,n-1}\right)}_{1}\right]}^{2}\right)p\left(F_{1:p}\right), \end{array}$$ (18)

with the prior *p*(*F*_1:*p*_) a structural prior which restricts sampling of *F*_1:*p*_ to the subspace of coefficients for which the process is stationary and its characteristic polynomial has *R* real roots and *C* pairs of imaginary roots. For what follows, we drop the pairs qualifier when referring to one of the pairs of imaginary roots. Instead of sampling the entire *F*_1:*p*_ at once, we sample one of the *C* imaginary or *R* real roots at a time while keeping the rest of the roots fixed, via Markov Chain Monte Carlo (MCMC) as follows.

The residuals of the AR(*p*) process are uncorrelated Gaussians, but leaving out one of the roots results in residuals that are correlated. We now outline the case when sampling an imaginary root. Here, we consider the correlated residual to be an AR(2) process. Ignoring the trial index, *x*_*n*_ is related to the *p* lagged values *x*_*n*−1_, …*x*_*n*−*p*_ through the AR coefficients and an innovation variance. Utilizing the backshift operator, $\hat{B}x_{n}=x_{n-1}$, eq (3b) can be written as a polynomial in $\hat{B}$, and the polynomial as a product of its *R* real roots *α*_*j*_, *j* ∈ {1, …, *R*} and *C* pairs of complex roots $\gamma_{j},\gamma_{j}^{*}$, *j* ∈ {1, …, *C*}.

With the roots calculated, the AR process, Eq (3b), can be represented as

$$\begin{array}{c} \epsilon_{n}=[\prod_{r}^{R}\left(1-\alpha_{r}\hat{B}\right)\prod_{c}^{C}\left(1-\gamma_{c}\hat{B}\right)\left(1-\gamma_{c}^{*}\hat{B}\right)]x_{n}. \end{array}$$ (19)

The coefficient for ${\hat{B}}^{i}$, *i* ∈ {1, …, *p*}, is *F*_*i*_. Regrouping the terms, we rewrite the residuals when the contribution of the *j*th complex root is removed, to obtain a new time series *ω*_*jn*_:

$$w_{jn}={\left(1-\gamma_{j}\hat{B}\right)}^{-1}{\left(1-\gamma_{j}^{*}\hat{B}\right)}^{-1}\epsilon_{n}=\prod_{r}^{R}\left(1-\alpha_{r}\hat{B}\right)\prod_{c\neq j}^{C}\left(1-\gamma_{c}\hat{B}\right)\left(1-\gamma_{c}^{*}\hat{B}\right)x_{n}.$$ (20)

This equation tells us how to obtain the *ω*_*jn*_, and that we are treating that time series as an AR(2). We note that given a timeseries **x** *_m_* and some *F*_1:*p*_, we could calculate the residual ***ϵ***, and this would be the same series of *N* numbers had we first obtained the **w** *_j_*, and then calculated the *N* $\epsilon_{n}=\left(1-\gamma_{j}\hat{B}\right)\left(1-\gamma_{j}^{*}\hat{B}\right)w_{jn}$.

Given that its eigenvalues $\gamma_{j},\gamma_{j}^{*}$ have corresponding angle 2*π*/λ_*j*_ and modulus *r*_*j*_, which control the frequency and the steadiness of the oscillation when these parameters are used to generate a time series. Using Vieta’s formula [34], the AR(2) coefficients $\phi_{1}=-\left(\gamma_{j}+\gamma_{j}^{*}\right)$ and $\phi_{2}=\gamma_{j}\gamma_{j}^{*}$ are related to the frequency and modulus as 2*r*_*j*_ cos(2*π*/λ_*j*_) and $-r_{j}^{2}$, and

$$\begin{array}{c} \phi_{j1},\phi_{j2}∼N\left(h,\frac{H^{-1}}{\sigma^{2}}\right) \end{array}$$ (21)

where

$$\begin{array}{ll} \begin{array}{rr} H_{j}^{-1}= & D_{j}^{T}D_{j},\\ h_{j}= & H_{j}D_{j}^{T}w_{j}^{\prime }, \end{array} & \begin{array}{ll} D_{j}^{T}= & \left(\begin{array}{cccc} w_{j0} & w_{j1} & \ldots & w_{j,N-1}\\ w_{j-1} & w_{j0} & \ldots & w_{j,N-2} \end{array}\right)\\ w_{j}^{\prime T}= & \left(\begin{array}{cccc} w_{j1} & w_{j2} & \ldots & w_{jN} \end{array}\right) \end{array} \end{array}$$ (22)

The stationary regime is defined by *r*_*j*_ ≤ 1, so in sampling from the conditional posterior, we must restrict the sampling to regions bounded by the curves

$$\begin{array}{c} \frac{\phi_{j1}^{2}}{4}+\phi_{j2}=0,\;\phi_{j2}=-1 \end{array}$$ (23)

This boundary describes a truncated 2-variate normal distribution.

When the root in question is real, we remove its contribution to obtain the correlated residuals

$$u_{jn}={\left(1-\alpha_{j}\hat{B}\right)}^{-1}\epsilon_{n}=\prod_{r\neq j}^{R}\left(1-\alpha_{r}\hat{B}\right)\prod_{c}^{C}\left(1-\gamma_{c}\hat{B}\right)\left(1-\gamma_{c}^{*}\hat{B}\right)x_{n}.$$ (24)

The real roots are can be sampled from a truncated univariate normal with mean *m*_*j*_ and variance *M*_*j*_

$$\begin{array}{c} m_{j}=\frac{\sum_{n=1}^{N}u_{jn}u_{j,n-1}}{\sum_{n=1}^{N}u_{j,n-1}^{2}},\;M_{j}=\frac{\sigma^{2}}{\sum_{n=1}^{N}u_{j,n-1}^{2}} \end{array}$$ (25)

We further assume a simple informative, uniformly distributed prior of [0.97, 1] for the modulus of the slowest root. Large modulus roots are spectrally peaked, and correspond to oscillations with a relatively well-defined frequency. Our choice of a prior on the modulus is similar to adding regularization terms to the likelihood to address concerns about overfitting when data is limited, an alternative avenue recently investigated by Buesing et al [35], although this requires the structured decomposition of the AR model, which would be difficult to express in terms of regularization functions.

#### The latent oscillation: X data augmentation

The predictive distribution for **X** can be read off from Eq (4), and is

$$\begin{array}{c} X∼\prod_{m}^{M}\left[\prod_{n=0}^{N}N\left(x_{mn}+f_{n}+\mu_{m}+\lambda_{l\left(m,n\right)}^{R}|\frac{\kappa_{mn}}{\omega_{mn}},\frac{1}{\omega_{mn}}\right)\prod_{n^{\prime }=1}^{N}p(X_{mn^{\prime }}|X_{m,n^{\prime }-1},\Theta )p(X_{m0}|\Theta )\right] \end{array}$$ (26)

The *mn*th observation, which through augmentation by the Pólya-Gamma variables, is no longer the spikes, but a continuous variable $t_{mn}=\kappa_{mn}/\omega_{mn}-f_{n}-\mu_{m}-\lambda_{l(m,n)}^{R}$ with observation noise *r*_*mn*_ = 1/*ω*_*mn*_. We generate a sample of **X** given observation **t** and fixed Θ by employing the FFBS (Forward-filter backward-sample) algorithm [30]. Briefly, FFBS is a recursive algorithm with the same forward filtering step as the Kalman Filter, but on the backward step, instead of obtaining the mean from the smoothing density, we obtain realizable sample paths of the latent state from the smoothing density. The *M* trials are independent, so for the sake of simple notation, we drop the trial index *m* in what follows. Forward filtering is done by recursive application of a prediction and filtering step.

$$\begin{array}{c} \begin{array}{cc} \text{prediction} & \text{filter}\\ {\hat{X}}_{m,n|n-1}=F{\hat{X}}_{m,n-1|n-1} & {\hat{X}}_{m,n|n}={\hat{X}}_{m,n|n-1}+K_{mn}\left(t_{mn}-H{\hat{X}}_{m,n|n-1}\right)\\ {\hat{V}}_{m,n|n-1}=F{\hat{V}}_{m,n-1|n-1}F^{T}+Q & {\hat{V}}_{m,n|n}=\left(I-K_{mn}H\right){\hat{V}}_{m,n|n-1} \end{array} \end{array}$$ (27)

with

$$Q=\left(\begin{array}{l} \begin{array}{ll} \begin{array}{c} \sigma^{2}\\ 0\\ \vdots \end{array} & \begin{array}{c} 0\ldots \\ 0 \end{array} \end{array} \end{array}\right),\;H=\left(1\;0\;\ldots \right)$$ (28)

and the Kalman gain, $K_{mn}={\hat{V}}_{m,n|n-1}H^{T}{\left(H{\hat{V}}_{m,n|n-1}H^{T}+r_{mn}\right)}^{-1}$. The prediction mean is straightforward given the structure of **F**, while the prediction covariance is

$$\begin{array}{c} {\hat{V}}_{m,n|n-1}=\left(\begin{array}{cc} \sum_{ij}F_{i}{\left({\hat{V}}_{m,n-1|n-1}\right)}_{ij}F_{j}+\sigma^{2} & {\sum_{i}F_{i}\left({\hat{V}}_{m,n-1|n-1}\right)}_{i,1\ldots p-1}\\ {\sum_{i}F_{i}\left({\hat{V}}_{m,n-1|n-1}\right)}_{1\ldots p-1,i} & {\left({\hat{V}}_{m,n-1|n-1}\right)}_{1\ldots p-1,1\ldots p-1} \end{array}\right) \end{array}$$ (29)

The Kalman gain becomes

$$\begin{array}{c} K_{mn}=\frac{1}{{\left({\hat{V}}_{m,n|n-1}\right)}_{11}+r_{mn}}\left(\begin{array}{c} {\left({\hat{V}}_{m,n|n-1}\right)}_{11}\\ \vdots \\ {\left({\hat{V}}_{m,n|n-1}\right)}_{p1} \end{array}\right), \end{array}$$ (30)

while filter mean is ${\hat{X}}_{m,n|n}={\hat{X}}_{m,n-1|n}+K_{mn}\left[t_{mn}-{\left({\hat{X}}_{m,n|n-1}\right)}_{11}\right]$ and covariance is

$$\begin{array}{c} {\left({\hat{V}}_{m,n|n}\right)}_{ij}={\left({\hat{V}}_{m,n|n-1}\right)}_{ij}-\frac{{\left({\hat{V}}_{m,n|n-1}\right)}_{1i}{\left({\hat{V}}_{m,n|n-1}\right)}_{1j}}{{\left({\hat{V}}_{m,n|n-1}\right)}_{11}+r_{mn}} \end{array}$$ (31)

FFBS requires starting values ${\hat{X}}_{m,0|0}$ and ${\hat{V}}_{m,0|0}$ for the recursion, for which we choose **0** and a reasonably broad, diagonal covariance, respectively. The inferred oscillation in the begin of the trials will be of poorer quality, but improves quickly after enough spikes are observed. After the last time point is reached, the latent state is sampled backwards in time. The smoothing distribution can be written

$$\begin{array}{c} p(X_{m,1\ldots N}|t_{m,1\ldots N},\Theta )\propto p(X_{N}|t_{m,1\ldots N},\Theta )\prod_{n=0}^{N-1}p(X_{mn}|X_{m,n+1},t_{m,1\ldots n},\Theta ), \end{array}$$ (32)

the first term a normal distribution about ${\hat{X}}_{m,N|N}$ with covariance ${\hat{V}}_{m,N|N}$, and the term under the product written in terms of known distributions

$$\begin{array}{c} p(X_{mn}|X_{m,n+1},t_{m,1\ldots n},\Theta )\propto p(X_{m,n+1}|X_{mn},\Theta )p(X_{mn}|t_{m,1\ldots n},\Theta ), \end{array}$$ (33)

which is a product of the transition density, which is a normal distribution with mean **Fx** *_mn_* and variance **Q**, and a filter density with mean ${\hat{X}}_{m,n|n}$ and variance ${\hat{V}}_{m,n|n}$. Defining $A\equiv {\hat{V}}_{m,n|n}F^{T}{\left(F{\hat{V}}_{m,n|n}F^{T}+Q\right)}^{-1}$, the sampling distribution of **X** *_m,n_* is a multivariate normal with mean and covariance given by

$$\begin{array}{ll} {\hat{X}}_{m,n|N}= & \left(I-AF\right){\hat{X}}_{m,n|n}+AX_{m,n+1}\\ {\hat{V}}_{m,n|N}= & \left(I-AF\right){\hat{V}}_{m,n|n}. \end{array}$$ (34)

**X**_*m*,*n*+1_ is the previously-sampled laten state **Q** can be expressed as an outer product of a column vector **u** = (σ, 0, 0, …) with itself, **uu**^*T*^. Using the Sherman-Morrison formula [36] for the rank-1 update of an inverse matrix, the moments of the backward-sampling densities may be written as

$$\begin{array}{cc} {\hat{X}}_{m,n|N}= & \left(\begin{array}{c} {\left(X_{m,n+1}\right)}_{2}\\ \vdots \\ {\left(X_{m,n+1}\right)}_{p-1}\\ F_{s}-\frac{\sigma^{2}{\left(F_{pi}^{-1}\right)}^{2}\left[F_{s}{\left(V_{m,n|n}^{-1}\right)}_{pp}+\sum_{i=1}^{p-1}{\left(X_{m,n+1}\right)}_{i+1}{\left(V_{m,n|n}^{-1}\right)}_{pi}-\sum_{i}^{p}{\left(V_{m,n|n}^{-1}\right)}_{pi}{\left({\hat{X}}_{m,n|n}\right)}_{i}\right]}{1+\sigma^{2}{\left(V_{m,n|n}^{-1}\right)}_{pp}{\left(F^{-1}\right)}_{p1}^{2}} \end{array}\right) \end{array}$$ (35)

$${\hat{V}}_{m,n|N}=\frac{1}{1+\sigma^{2}{\left(V_{m,n|n}^{-1}\right)}_{pp}{\left(F^{-1}\right)}_{p1}^{2}}\left(\begin{array}{cc} 0 & \begin{array}{l} 0\\ \vdots \end{array}\\ 0\;\ldots & \sigma^{2}{\left({\left(F_{-1}\right)}_{p1}\right)}^{2} \end{array}\right)$$ (36)

where $F_{s}\equiv \sum_{i=1}^{p}{\left(F^{-1}\right)}_{pi}{\left(X_{m,n+1}\right)}_{i}$. The AR(*p*) model with the extended state-space is only partly dynamic [30], with rank (**Q**) = 1 < *p*, and the computations necessary for the FFBS equations are much less than would be necessary for a full-rank model, as can be seen from the calculations of various moments presented in this section.

#### Pólya-Gamma data augmentation

The predictive distribution for *ω*_*mn*_, the term under the integral marginalizing out *ω*_*mn*_ in Eq (8), is the general *PG*(*ω*_*mn*_|*b* = 1, *z* ≠ 0) distribution, obtained by an exponential tilting of the PG(*ω*_*mn*_|*b* = 1, *z* = 0) distribution [25]:

$$\begin{array}{c} \omega_{mn}∼PG\left(\omega_{mn}|1,\psi_{mn}\right)=2^{-1}e^{\kappa \psi_{mn}}e^{-\frac{\omega_{mn}\psi_{mn}}{2}}PG\left(\omega_{mn}|1,0\right), \end{array}$$ (37)

with $\psi_{mn}=x_{mn}+f_{n}+\mu_{m}+\lambda_{l(m,n)}^{R}$. This is sampled using the rejection sampler described in Polson *et al* [25].

#### Inferring additional latent structures

Oscillations temporally structure the spike train, but it is possible that these oscillations do not occur uniformly throughout all trials. The LOST model allows inferring additional structure present in the neural data, such as modulation of oscillatory strength within a trial, or trends in modulation strength across trials. In the simulation studies, we consider an example where the spike train is significantly modulated in only a fraction of the trials, and demonstrate that the LOST model is able to identify these trials. We also apply this to real data to show the LOST model can predict, using only spike train data, which trials are strongly modulated to a theta rhythm in the LFP, and which are not. For these applications, we assume the modulation strength takes on 2 distinct values. We fix the modulation strength of unmodulated trials to *s*_0_ = 0.1, and parameterize the modulation strength of the modulated trials, *s*_1_. We introduce the latent indicator variable **Z**, which is an (*M* × 2) matrix where, for example, **Z**_*m*_ = (1, 0) if the *m*th trial is an unmodulated trial, and also the parameter mixture weights ***π***, a 2 component column vector whose components sum to 1. The likelihood is then

$$\begin{array}{lll} p(y|\Theta ) & = & \int_{X}\prod_{m}\sum_{Z_{m}}p(y_{m},Z_{m}|X_{m},\Theta )p(X_{m}|\Theta )dX\\ & = & \int_{X}\prod_{m}\sum_{Z_{m}}\prod_{j}{\left[\pi_{j}p_{j}(y_{m},{|X}_{m},\Theta )\right]}^{Z_{mj}}p(X_{m}|\Theta )dX\\ & = & \int_{\text{X}}\prod_{m}\sum_{{\text{Z}}_{m}}\prod_{j}{\left[\pi_{j}\prod_{n}Ber(y_{mn},|x_{mn},Z_{mj},\Theta )\right]}^{Z_{mj}}p({\text{X}}_{m}|\Theta )d\text{X}\\ & = & \int_{\text{X}}\prod_{m}\sum_{{\text{Z}}_{m}}\prod_{j}[\pi_{j}\times \prod_{n}{\int N\left(s_{j}x_{mn}+f_{n}+\mu_{m}+\lambda_{l(m,n)}^{R}|\frac{\kappa_{mn}}{\omega_{mn}},\frac{1}{\omega_{mn}}\right)p(\omega_{mn})d\omega_{mn}]}^{Z_{mj}}p({\text{X}}_{m}|\Theta )d\text{X} \end{array}$$ (38)

Define *M*_*L*_ to be all *m* such that **Z**_*m*_ = (1, 0) and *M*_*H*_ to be all *m* such that **Z**_*m*_ = (0, 1). The likelihood contribution to the conditional posterior for *s*_1_ is Gaussian, $s_{1}∼N(s_{1}|\frac{B}{2A},\frac{1}{2A})$, where

$$\begin{array}{c} A\equiv \sum_{m\in M_{L},n}\frac{\omega_{mn}x_{mn}^{2}}{2}\;B\equiv \sum_{m\in M_{L},n}\omega_{mn}x_{mn}(\frac{\kappa_{mn}}{\omega_{mn}}-f_{n}-\mu_{m}-\lambda_{l(m,n)}^{R}). \end{array}$$ (39)

For the prior distribution for *s*_1_, we use $p\left(s_{1}\right)=N\left(s_{1}|\mu_{s_{1}}\sigma_{s_{1}}^{2}\right)$, where $\mu_{s_{1}}=s_{0}$, and a large variance $\sigma_{s_{1}}^{2}$, because we have no prior belief about what *s*_1_ should be. The data augmentation of **X** analogous to Eq (26) is carried out by replacing the *mn*th observation and noise by

$$\begin{array}{c} t_{mn}\to \frac{\kappa_{mn}/\omega_{mn}-f_{n}-\mu_{m}-\lambda_{l\left(m,n\right)}^{R}}{\prod_{j}s_{j}^{z_{mn}}}\;r_{mn}\to \frac{1}{{\left(\omega_{mn}\prod_{j}s_{j}^{z_{mj}}\right)}^{2}}. \end{array}$$ (40)

The Pólya-Gamma data augmentation analogous to Eq (37) is carried out by replacing the *mn*th *ψ*_*mn*_ by

$$\begin{array}{c} \psi_{mn}\to \prod_{j}s_{j}^{Z_{mj}}x_{mn}+f_{n}+\mu_{m}+\lambda_{l(m,n)}^{R}. \end{array}$$ (41)

The data augmentation for *Z*_*mj*_ is done by generating from a 2-category multinomial distribution. The probability of the *m*th trial being in the *j*th category is proportional

$$\begin{array}{c} p(Z_{mj}=1|\Theta ,\;V^{+})\propto \pi_{j}\underset{n}{\prod }\frac{{\left(e^{s_{j}x_{mn}+f_{n}+\mu_{m}+\lambda_{l\left(m,n\right)}^{R}}\right)}^{y_{mn}}}{1+e^{s_{j}x_{mn}+f_{n}+\mu_{m}+\lambda_{l\left(m,n\right)}^{R}}} \end{array}$$ (42)

The ***π*** are sampled from a Dirichlet distribution

$$\begin{array}{c} \pi ∼Dir(\alpha_{\pi ,0}+\sum_{m\in M_{L}}Z_{m1},\alpha_{\pi ,1}+\sum_{m\in M_{H}}Z_{m2}). \end{array}$$ (43)

We set the vector ***α*_*π*_** = (1, 1).

### Numerical computation

Gibbs samplding was implemented in the Python programming language using the Numpy, SciPy, Matplotlib, StatsModels, and Patsy toolboxes. Numerical routines were written in C/C++ and Cython. Software for the Gibbs sampler and a Python wrapper for the Pólya-Gamma routine based on the original code by Jesse Windle, available at https://github.com/jwindle, is available at https://github.com/AraiKensuke/PP-AR and https://github.com/AraiKensuke/pyPG, respectively.

## Results

To illustrate how the LOST model can infer latent oscillations under a variety of realistic conditions, we simulated biologically plausible oscillations, and spike trains modulated with these oscillations using either a simple inhomogeneous renewal process, or the leaky integrate-and-fire (LIF) neuron model. Further, we describe another point process method to infer the CIF, the GLM method, for comparison with the LOST model.

### Simulated oscillations and spike trains

**Fig 1:**
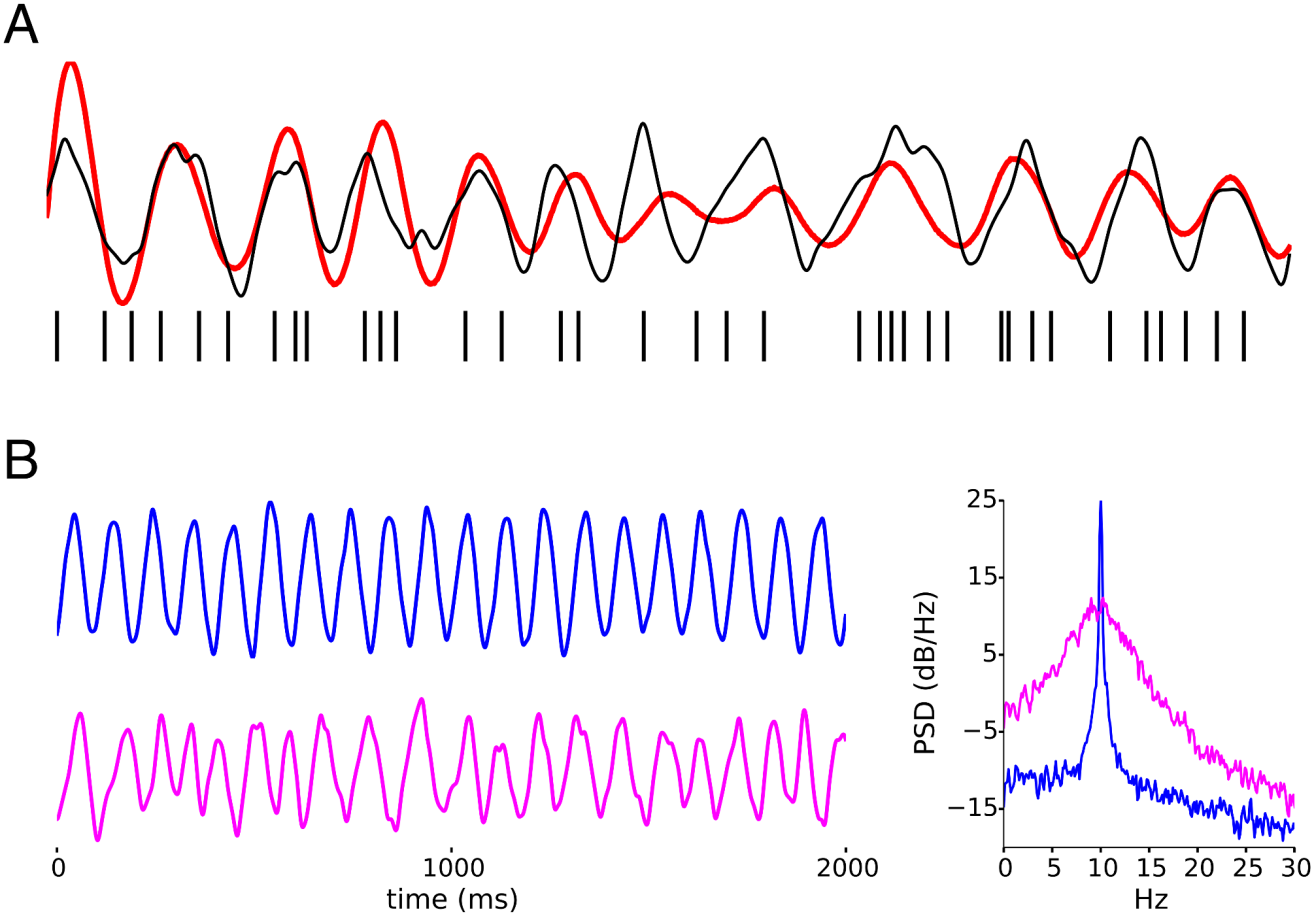
Regular and irregular oscillations. A) LOST infers (red) the oscillatory modulation (black), if any, underlying the spikes (vertical ticks) in a spike train, using only the spiking data. The oscillation amplitude stabilizes after the first few spikes are observed. B) We refer to spectrally narrow (wide) oscillations as regular (irregular), shown by the blue (pink) oscillation with OCV = 0.02 (0.22). The correlation between the current and past phase decays slowly (quickly) as a function of lag in regular (irregular) oscillations.

The stochastic oscillation for the *m*th trial **w**_*m*_ is generated by first generating a stochastic phase **t**_*m*_ and stochastic amplitude **A**_*m*_ as

$$\begin{array}{cc} t_{m,n+1}=t_{mn}+\Delta t(1+\frac{\xi_{mn}}{C_{\xi }})\;w_{mn} & =1+\frac{A_{mn}}{C_{A}}\sin\left(2\pi \nu t_{mn}\right). \end{array}$$ (44)

where ***ξ***_*m*_ and **A**_*m*_ are generated by AR(1) processes, and *t*_*m*0_ ∈ [0, 1] a uniform random number. These are then used to generate the irregular oscillation *C*_*ξ*_ and *C*_*A*_ control the size of deviation from uniformity, while the timescale of the AR(1) processes controls the timescale of variation of amplitude and phase in the oscillation. AR(1)s with timescales on the order of the oscillation period *T* = 1/*ν* produce fluctuations that causes considerable variability of period and amplitude in **w**, which we quantify with the oscillator irregularity, the oscillatory coefficient of variation (OCV) of the instantaneous periods *T* of oscillation, defined as the ratio of the standard deviation to the mean of time intervals between phase 0 crossings of an oscillatory signal. Fig 1 shows 2 example oscillations generated by this procedure, their OCVs and their power spectral densities. Irregular oscillations have a higher OCV and a less peaked spectral density.

To generate spikes, we employ an inhomogeneous renewal process or the LIF model, driven by a stochastic model of oscillation to modulate the firing of spikes. For the inhomogeneous renewal process, for each trial *m* and time *n*, if for a uniform random number *r*_*mn*_ ∈ [0, 1], $r_{mn}<\Delta t\left(e^{\mu_{m}+w_{mn}+\lambda_{l(m,n)}^{G}}\right)$, we generated a spike, with $\lambda_{l(m,n)}^{G}$ the user-defined ground truth refractory history function. For the LIF model, spikes were generated using

$$\begin{array}{cc} \Delta V_{mn} & =(-\frac{V_{mn}}{\tau }+f_{n}+\mu_{m}+w_{mn}+\chi_{mn})\Delta t, \end{array}$$ (45)

where the Gaussian random variable $\chi_{mn}∼N\left(0,\sigma_{\text{b}}^{2}\right)$ represents irregular arrival times of afferent spikes, *τ* = 0.2 the membrane time constant, *μ*_*m*_ a baseline DC current and $\sigma_{\text{b}}^{2}=210$ the amplitude of background fluctuation. Spikes are elicited when *V*_*mn*_ ≥ 1 passes the threshold value of 1, which then causes a reset to *V*_*m*,*n*+1_ = 0. LIF have a refractory period that depends on the model parameters, and therefore the CIF naturally is dependent on the last spike time.

#### LIF neuron with stimulus-triggered change in firing rate

**Fig 2:**
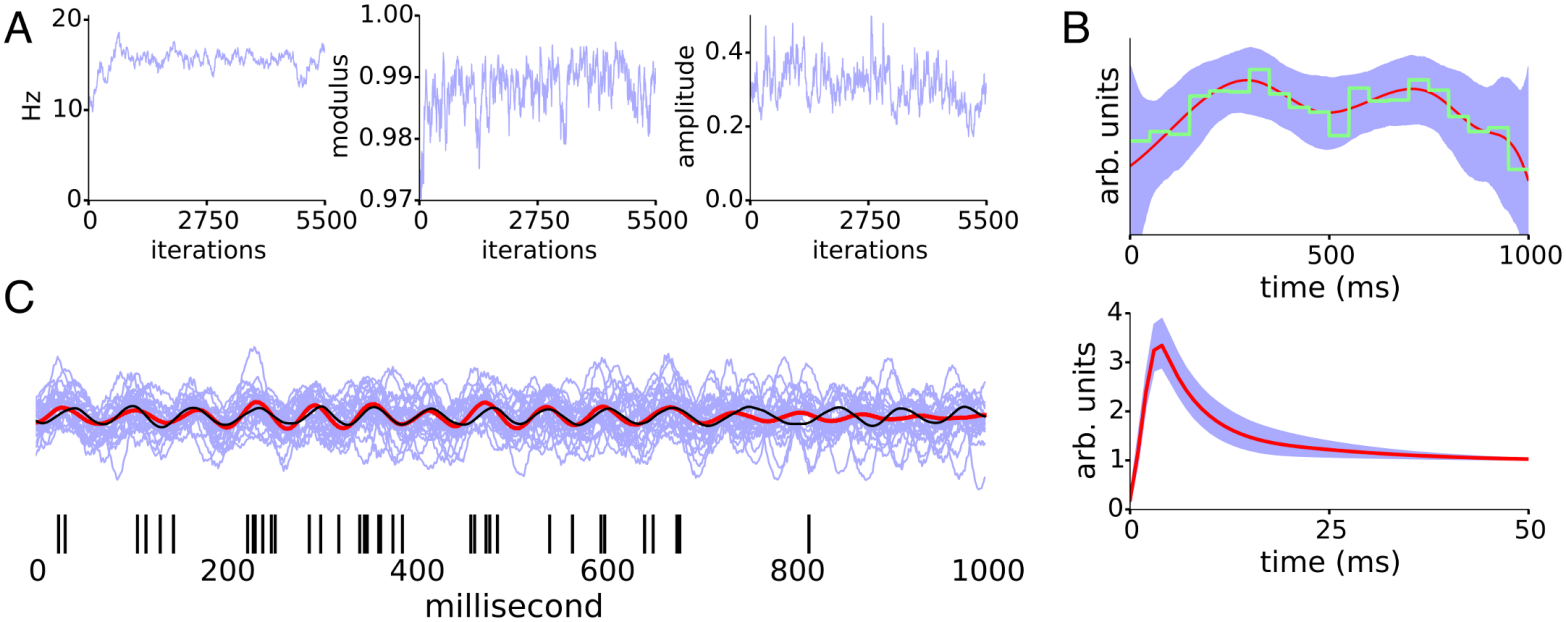
LIF neuron with stimulus-triggered change in firing rate. Phase inference for simulated LIF neuron driven by irregular oscillation with an average firing rate of 40Hz and a 15Hz modulating oscillation with OCV of 0.13. 60 trials of 1 second duration were used for LOST. A) Samples of the representative AR parameters. B) Top, samples of the TAE (cyan), the mean (red), and the empirical PSTH used to initialize TAE knot locations (green), and bottom, samples (cyan) of the spike history and mean (red). C) Example of inferred latent oscillation. The RL of the inferred oscillation is 0.65 ± 0.02, while the spike train has an *R* of 0.22 ± 0.01 with the ground truth oscillation.

We demonstrate the basic operation of LOST and the inference of the TAE, spike history and oscillation. In the following, we monitor a small subset of the full joint posterior parameters: the modulus *r*, frequency 2*π*/λ of the lowest AR(2) component, and the latent state amplitude defined by its standard deviation. Samples of trial offsets or the TAE knots approach their stationary distribution values much more quickly than these parameters representative of the latent state, so monitoring this smaller subset suffices for determining when the posterior LOST draws from has reached its stationary distribution. Fig 2A shows a clear oscillation being inferred, as the parameters show little uncertainty. We took the mean of the last 2000 iterations to calculate the means in Fig 2B, and the oscillation in Fig 2C. In most of the following figures, we use the convention that cyan represents sampled values themselves, their distribution or 5-95% intervals of the sampled values. Red lines represent means of sampled values, and black lines represent ground truth values.

#### Model identifiability and interpreting the results of LOST

**Fig 3:**
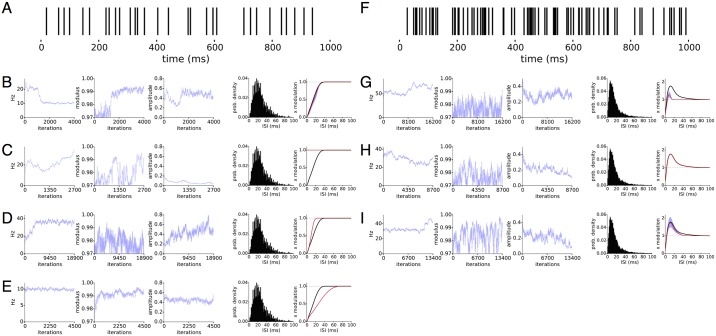
Interaction of spike history and latent state. Two inhomogeneous, history dependent spike trains, with first spike train A) generated with stochastic oscillational modulation at 10Hz and OCV at 0.18, and spiking at 40Hz, and the second spike train F) without modulation, spiking at 60Hz. Gibbs samples from 4 (3) separate LOST for first (second) spike train in B-E (G-I). In B-E and G-I, we show the 3 representative sampled quantities, frequency and modulus of the slowest root and the amplitude of the inferred latent state on the left, and the ISI distribution and spike history term on the right. The ground truth, sampled mean and 5-95% intervals of the sampled spike history term plotted in black, red and cyan, respectively. The differences between the different LOST runs is in how spike history knots were chosen, or if a fixed spike history function was used. For the first spike train A, B uses the standard knot choices, C uses a constant history set to 0 and D, a fixed spike history set to a time-compressed ground-truth history. For the second spike train, G uses the standard knot choice, H the ground truth and I the standard knots moved farther out in time. For the first spike train, misspecification of the history may lead to a false inference of a flat latent state, a latent state with high uncertainty in its parameters or the correct latent state. For the second spike train, a misspecified history can lead to a latent state with significant amplitude but with uncertain parameters, making the inference inconclusive.

Oscillations and post-spike refractory periods both add temporal structure to spike trains. Because they are presumably separate biological mechanisms and modify the spike train in distinct ways, we model them separately using the latent state and the spike history term. Without imposing constraints on these two terms, the latent state and spike history cannot, in principle be separated. LOST imposes constraints on the latent state and spike history according to our prior beliefs about their properties. The knot locations impose a strong prior on the shapes the spike history can take, so misplacing the knots may strongly affect the inferred latent state, presenting a significant difficulty in interpreting the results. We therefore investigate how mis-specifying the history affects LOST in Fig 3, which shows typical results from mis-specifying the spike history with two simulated datasets. The datasets are spike trains generated by a renewal process, Fig 3A with and Fig 3F without oscillatory modulation.

For the first spike train, the standard spline knot locations allows the successful inference of an oscillation and the history, Fig 3B, as the frequency, modulus and amplitude conditional posteriors are all very certain. The use of a fixed, flat history results in a flat latent state, Fig 3C, as the pronounced silences immediately following spikes are increasingly unlikely under spike train models with the latent state alone, which tends to increase the firing rate immediately following a spike. The latent states take large amplitudes near the spiking frequency of 40Hz when a temporally-compressed version of the ground truth spike history is used, Fig 3D. While the frequency has certainty, the modulus and amplitudes in contrast have large uncertainty, lowering our confidence that this latent state represents an oscillatory modulation. It is likely that a wide range of latent model parameters are nearly interchangeable, leading to a widening of the posterior in the direction of the AR parameters. Stretching the spike history, Fig 3E, results in a similar inferred latent state as Fig 3B, so a mis-specified history does not always lead to spurious results. We analyzed the second spike train 3 times with LOST, twice with an adjustable spike history term but with different history knot locations, and once with a fixed spike history term set to the ground truth, and Fig 3G–3I show samples from their respective posteriors. Fig 3G uses knots chosen by changing the default knot finder parameters, and the amplitudes do not go to 0 while the uncertainty in AR parameters remain high. Fig 3H uses the fixed ground truth history, and the amplitude approaches 0, as it does in Fig 3I where knots were placed farther out than in Fig 3G, and the amplitude also approaches 0. We conclude that if the post-spike inhibition is not well characterized, the inference of an oscillation will most likely fail. If the inhibition is well characterized but the rest of the history is not, we find either that an oscillation will still be inferred successfully, or that the posterior will be broad in some of the directions of the AR parameters, making it difficult to conclusively determine that the latent state represents an oscillation.

**Fig 4:**
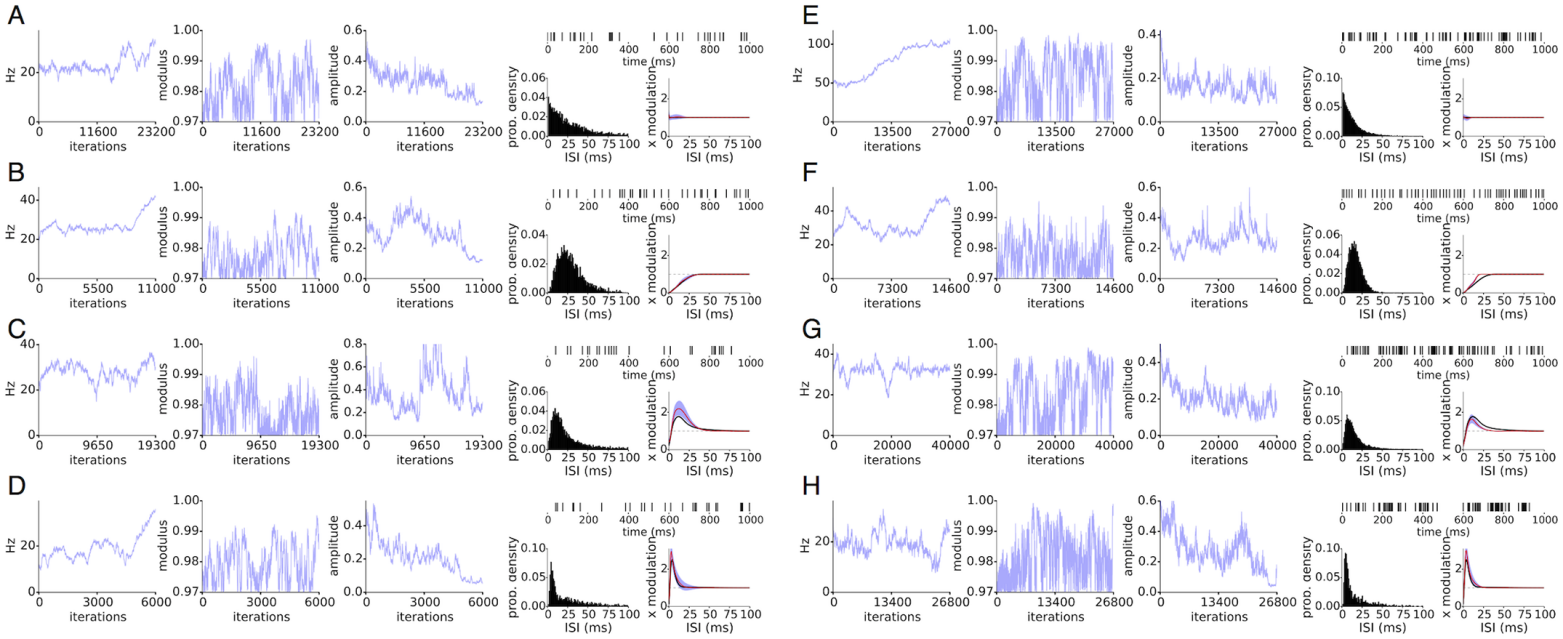
Spike trains without oscillatory modulation. Simulated spike trains at 34Hz (A-D) and 60Hz (E-H), with 4 types of post-spike refractory profiles. Each panel shows Gibbs samples on left and on the right, an example spike train at the top, and the ISI histogram and spike history function as in Fig 1. The expected output of LOST is a nearly flat latent state, which is the case in A, B, D, E, G, H, accompanied by large variability in the other sampled parameters. In C and F, the amplitude of the latent state does not approach 0, but rather continues to fluctuate while taking larger values.

We now look at spike trains without oscillational modulation, but generated with various post-spike refractory profiles to check that certain refractory profiles are not confounded as oscillations. Fig 4A–4D and 4E–4H are spike trains with average firing rate around 34Hz and 60Hz, respectively. In all cases except Fig 4C and 4F, the latent state amplitude approaches 0 as expected. For Fig 4C and 4F, the amplitude does not approach 0 but fluctuates widely along with the other parameters. In these cases, the large uncertainty in the AR parameters suggests the results of LOST are ambiguous, and we refrain from drawing a conclusion. We find that a reasonable rule of thumb in assessing how much uncertainty is “too much” is that frequency 2*π*/λ has a standard deviation of around 10% of its mean or less, and that modulus *r* have a standard deviation of under 0.005. Typically, we also find slow trends over a few thousand Gibbs iterations in the sampled values is commonly observed, consistent with the view that the parameters are nearly interchangeable over a fairly broad region of parameter space and the Gibbs sampler then performs a near random walk over the parameters. A latent state with a large amplitude does not necessarily signify the successful inference of an oscillation, and the uncertainty in the model parameters of the oscillation must also be taken in account in deciding whether to reject the hypothesis that there is an oscillation modulating the spiking. The amplitude of the latent state can inform us of how large the modulation is surrounding the mean firing rate. A latent state amplitude of 0.15 equates to a peak-to-peak fluctuation of about 30% of the average firing rate (for low firing rates < 100Hz), which is quite small a fluctuation considering only a few spikes may be observable for an oscillational cycle, so we use a latent state amplitude of 0.15 as a rule of thumb for when to consider the latent state as practically flat.

**Fig 5:**
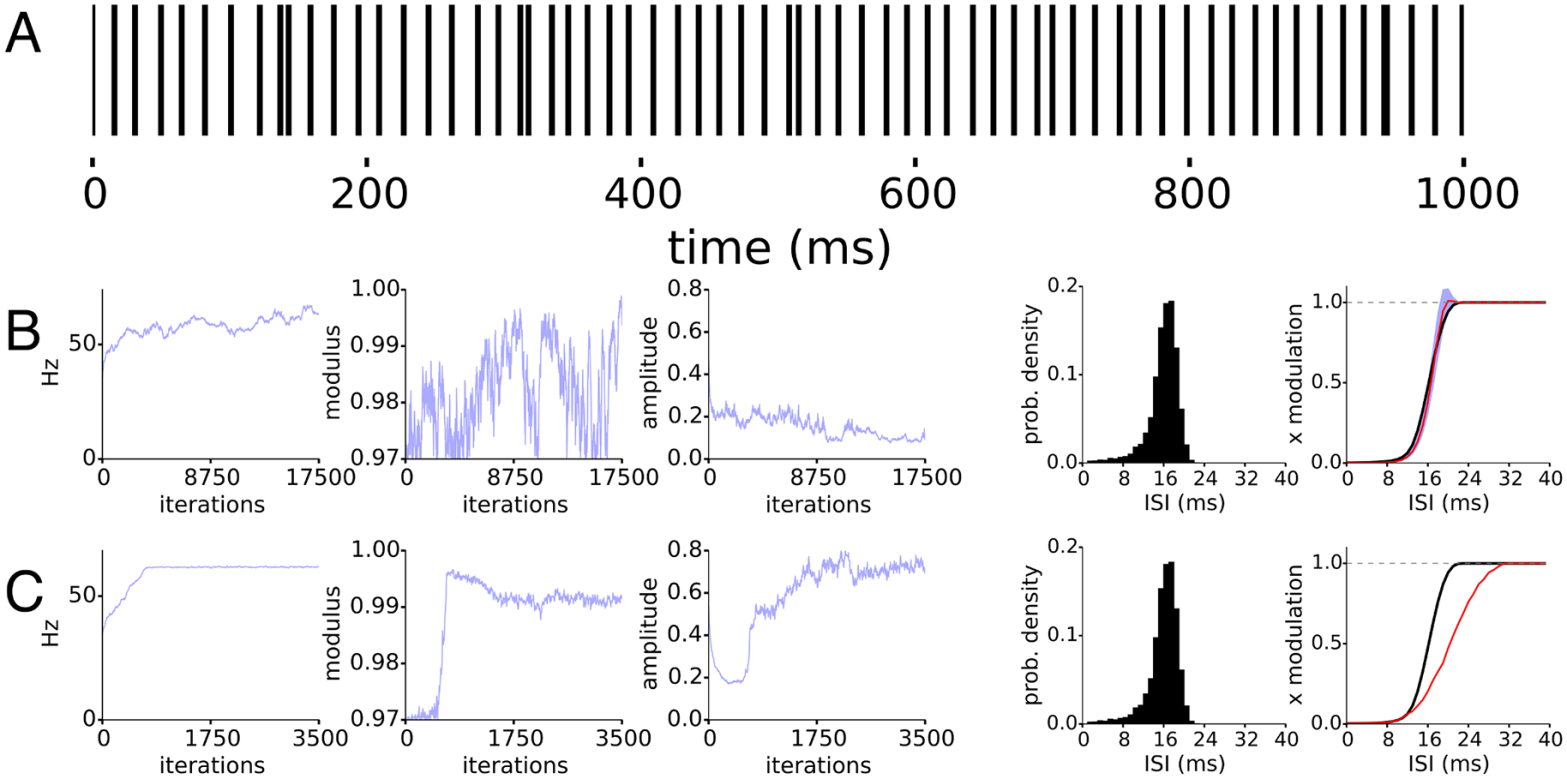
A) limiting case where models are not identifiable. The spike train in A is nearly periodic, but generated without an oscillatory modulation. When using the standard knots, the latent state amplitude approaches 0, B, but when we use a fixed history that is very different than the ground truth, C, the sampled parameters appear to be very certain, and an oscillation at 62Hz, nearly the spiking frequency, 64hz, is inferred. For cases where the spike train itself is nearly periodic, the spike train may be described equally well with various combinations of spike histories and an oscillation at the spiking frequency.

Through our examples, we found that the latent state can partially compensate an incorrectly specified spike history, as long as the short-term inhibition is reasonably well characterized by the spike history, or the post-spike inhibition is not too pronounced or long. In the absence of oscillational modulation in the data, this causes the latent state to take on significant amplitude, but the AR parameters themselves also have large uncertainty, making it difficult to conclude that the latent state represents an oscillation. However, in one limiting case, even with a mischaracterized spike history, LOST infers a clear oscillation. A nearly periodic spike train in Fig 5A is shown analyzed with LOST using the adjustable spike history term, Fig 5B and a fixed but modified ground truth spike history term, Fig 5C. When the misspecified history is used, the latent state takes large amplitudes with the posterior distribution of AR parameters having little uncertainty. The frequency of oscillation is nearly the same as the spiking frequency, 62Hz. In the limit of nearly periodic spikes, it may be difficult to conclude what particular combination of latent state and spike history underlies the highly regular spiking.

**Fig 6:**
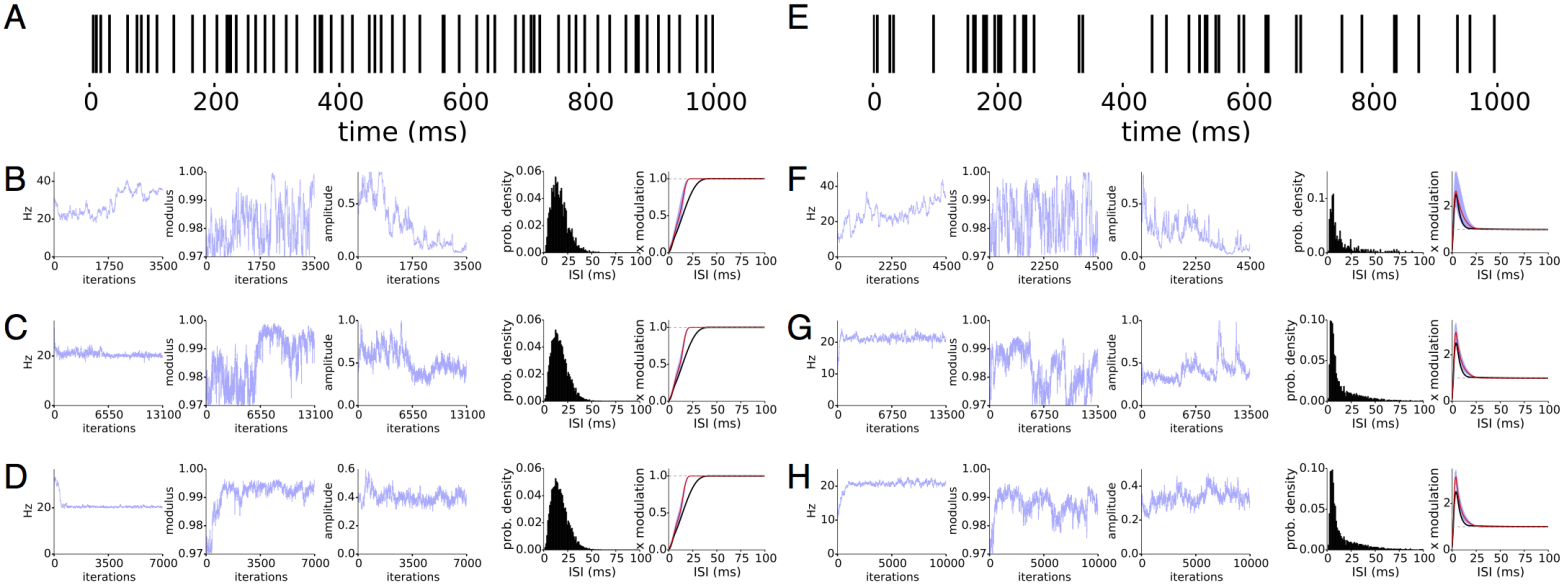
Effect of data size on posterior samples. 2 simulations of spike train with spiking around 60Hz, modulated with a 20Hz oscillation with OCV around 0.14 and 1 second long trials, but with different refractory period profiles, A and E. Layout of the figures is the same as in Fig 3, but with B-D and F-G showing different number of trials used, B) 8, C) 15 trials, and D) 40 for spike train in A and F) 5, G) 60 and H) 80 trials for spike train in E. For the smallest number of trials, the posterior is noticeably more variable, but the amplitude of the latent state approaches 0, meaning the latent state is almost decoupled from the spiking. We can see that the posterior narrows as more trials are used.

A flat latent state may signify a lack of oscillatory modulation or a spike history term that poorly captures the post-spike inhibition, or it may also signify that there is not enough data to infer an oscillation. Two spike trains with oscillatory modulation and different refractory profiles are shown in Fig 6A and 6E, with differing amount of trials used in LOST analysis, Fig 6B–6D and 6F–6H. When there is insufficient data to detect an oscillation, the latent state amplitude approaches 0, Fig 6B and 6F. In general, the uncertainty in the parameters of the oscillation will decrease as more trials and data are used, Fig 6C, 6D, 6G and 6H, as expected.

#### Model comparison

We compare the LOST model to another point process model whose CIF is a general linear model (GLM) of past spiking history [12, 18]. Briefly, the CIF for trial *m* time *n* is written

$$\begin{array}{c} \lambda \left(m,n|H_{m,n}\right)=\exp(\sum_{i=1}^{S}{\bar{a}}_{i}y_{m,n-i}+\sum_{j=1}^{O}{\bar{b}}_{j}C_{mj}),\;C_{mj}=\sum_{k=Nj}^{(S+1)j-1}y_{m,n-k}, \end{array}$$ (46)

and this is easily fit to obtain the $\bar{a}$ and $\bar{b}$ coefficients. The $\bar{\text{a}}$ models the short-term refractory self-history and $\bar{b}$ encapsulates longer-term oscillatory self-history. This model does not take into account the dynamics of oscillation. Rather the spiking activity up to *S*(*O* + 1) steps in the past uniquely determine the firing probability at the present, in contrast to our model. We fit the model several times with different initial conditions and values of *S* and *L*, using the python function statsmodels.api.GLM, and compare their goodness of fit via the time-rescaling theorem [37], and find the best combination of *S* and *O*. We then calculate the CIF from that model. Interpreting the fluctuating CIF as an oscillation, we extract the instantaneous phases, and compare to the phases inferred by the LOST model. The GLM model does not separate out the contribution of the short-term refractory self-history and the longer-term oscillatory self-history to the CIF, so we attempted to extract the oscillatory contribution by low-pass filtering the obtained CIF. We refer to this method of inference as GLM hereafter.

We compare the quality of inference of different models by comparing the resultant length of their inference with the ground truth. The resultant length (RL) *L* is calculated as $Le^{i\Phi_{L}}=\frac{1}{N}\sum_{i}^{N}e^{i(\phi_{j}-\psi_{j})}$, where the ***ψ*** (***ϕ***) are the ground truth (inferred) phases, and is the circular equivalent to the mean squared error (MSE) of the ground truth and inferred phase. We also use another simple measure of characterizing the strength of oscillatory modulation, the spike phase (SP) histogram and its circular statistic *R*, calculated as $Re^{i\Phi }=\frac{1}{N}\sum_{n=1}^{N}e^{i2\pi \phi_{t_{n}}}$, where *t*_*n*_ is the time of the *n*th spike. Here *R* characterizes non-uniformity in the distribution of $\phi_{t_{n}}$. While they are calculated in a similar fashion, the RL directly compares the inferred phase with the ground truth or LFP, while the SP *R* measures the uniformity of the probability of spiking with respect to ground truth or LFP phase. We caution that a result *L* < *R* does not mean that the LOST model performed inference poorly. SP histogram detects modulation, but knowledge that spiking occurs at preferred phases does not also mean we know how to assign instantaneous phases given the spikes in a spike train. The error on *R*s and *L*s is calculated by bootstrap sampling with replacement of trials.

#### Comparison to other models of oscillation

**Fig 7:**
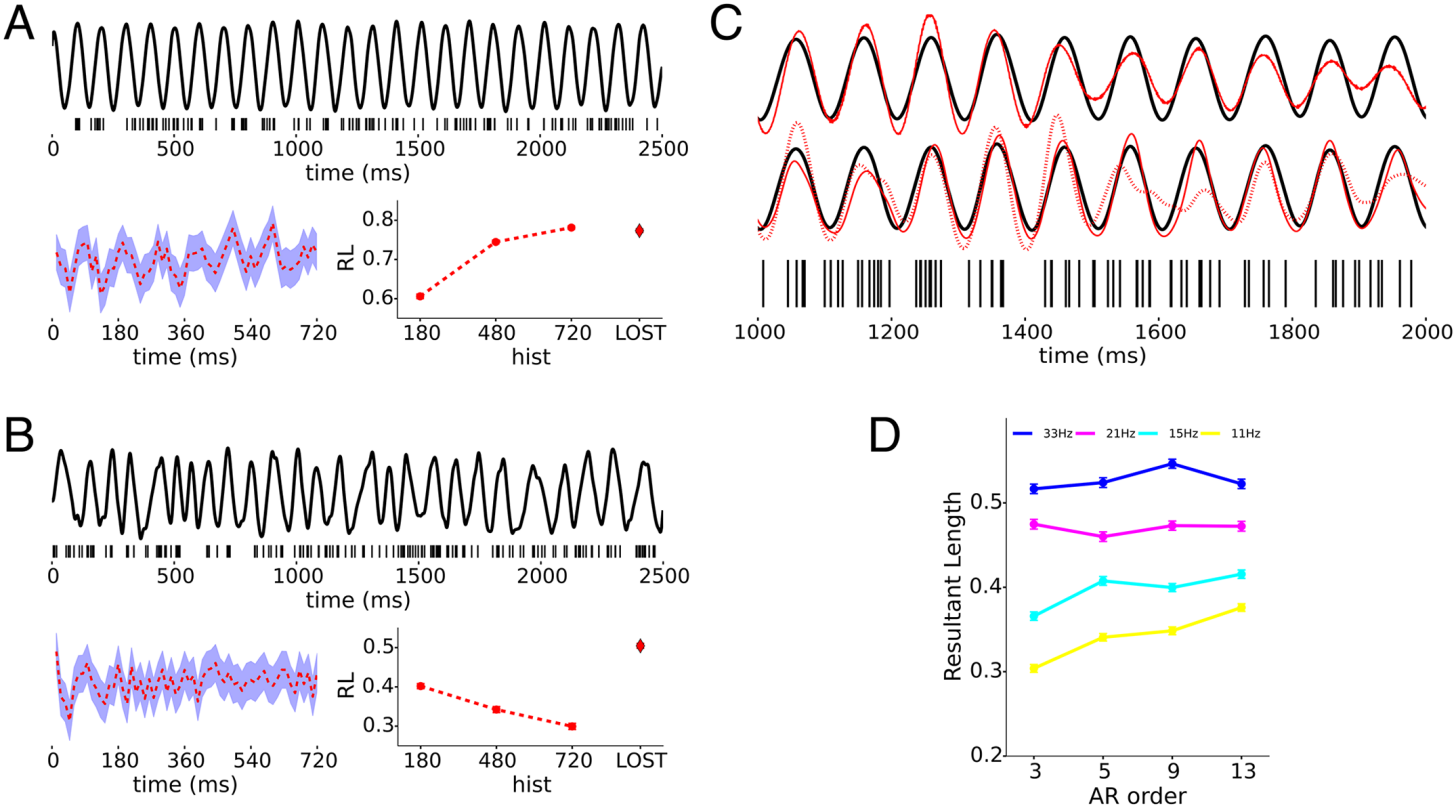
Comparison with other models of spiking and the AR order. Spiking noise and oscillation irregularity affect the quality of phase inference. A, B) Clockwise from top, spike train (ticks) produced by the regular oscillation plotted above, the resultant lengths when oscillation inferred using 3 different history lengths using GLM along with the RL from LOST inference of same data, and the history weights **b** (dashed red) and their confidence intervals (purple) for GLM using history length 720ms. Modulating oscillations have OCV = 0.02 and 0.2 in A) and B), respectively. C) Sample CIF inference of data shown in A) using LOST (top trace), and GLM (bottom trace), with black line the duplicated ground truth CIFs. For GLM, the dashed (solid) line is the inference obtained from using 720ms (180ms) of spiking history. D) Comparison of how different AR orders for the LOST model affect inference performance for irregular oscillations.

Comparison of the LOST model with the GLM model illustrates how point process noise and oscillatory irregularity pose challenges to inferring an oscillation from a spike train, and how explicitly modeling the dynamics of the oscillation using a latent state with a point process observation model, improves inference when posed with these challenges. Fig 7A and 7B show the dependence of RL on how much spike history is used in the GLM (bottom right), and the history weights **b** when 180, 480 or 720ms of history is used. The oscillation OCVs were 0.02 and 0.2 for Fig 7A and 7B, respectively. Temporal phase relationship is preserved for large time lags, as can be seen in the clear periodicity of the history weights **b** in Fig 7A, and the GLM model performs as well as the LOST model when long history dependence is used. Shorter history degrades the performance of the GLM model, because we are not averaging out the spiking noise as much when shorter history is used. However, Fig 7B shows that when the oscillation is irregular, the phase information quickly dissipates, as seen by the lack of periodicity in **b** at longer lags. Consequently, the spiking noise cannot be averaged out using long history, but rather spikes with large lag contribute only noise as the oscillational irregularity washes out the phase relationship for longer lags. The LOST model suffers less because it models the statistics of the irregularity in oscillation, the observable of the latent oscillation has a point-process observation model that takes into account spiking noise, and also because the inferred oscillation is conditioned on all the spikes in the trial, Fig 7C.

#### AR order

Huerta [24] describes a reversible jump MCMC procedure to choose an appropriate AR model strucure. We did not implement this due to the complexity of implementation and the relatively high computational cost of the current algorithm. Nonetheless, we investigate the effect of varying the AR order for phase inference with the LOST model. In Fig 7D, we used 4 sets of data with increasing firing rates, and compared the RLs of inference with different AR orders, and the accompanying RL from inference using GLM. The data sets themselves are generated with an oscillation of 10Hz using the LIF model, with OCVs of around 0.2, and have mean firing rates of 11Hz, 15Hz, 21Hz and 33Hz. 140, 130, 100, and 90 trials of 1.2 seconds duration were used in the fitting of the data. The model order comparison in Fig 7D shows that including more components usually resulted in a better inference of instantaneous phase, but qualitatively, even few components can pick out the oscillatory structure present in the spike train. This suggests that a model with the correct order, while important for optimal inference, can still infer valuable information if a sub-optimal order is chosen. For the rest of the paper, we keep the model structure to 4*C* + 1*R*, unless otherwise stated, as it seems to provide a good compromise between good inference and reasonable computation times.

#### Presence of additional, slower fluctuation

**Fig 8:**
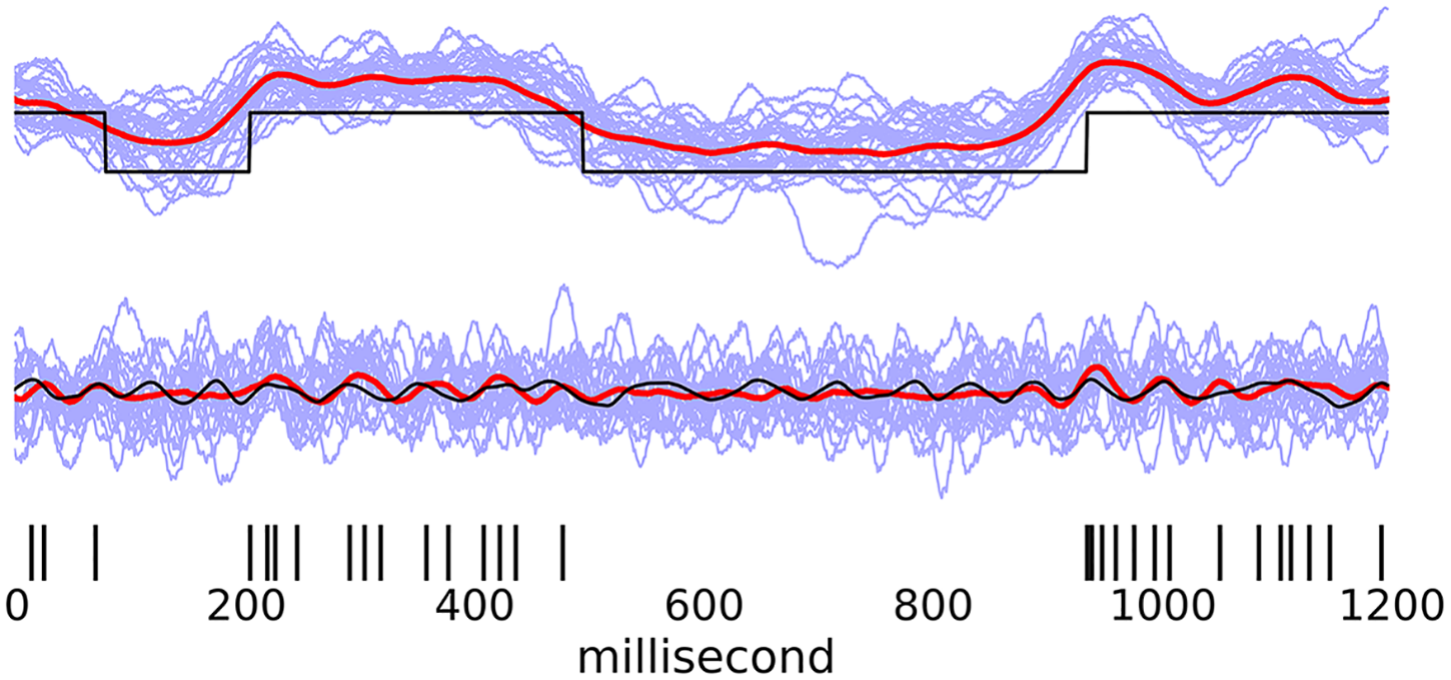
Spike train modulated with fast oscillation and a slower square-wave fluctuation. For a sample trial, top shows inferred, square-wave modulation of spiking (black) and the inferred latent state (red). Bottom shows inference of faster rhythm by taking the slow signal shown above to be a known signal, and re-running LOST.

Periods of prolonged silence, followed by prolonged periods of elevated firing rates are often visibly apparent in spike trains [38]. The timescales of these readily visible features are much slower than those characterizing faster neuronal oscillations thought to be relevant to cortical processing. In this example, we seek to demonstrate that the LOST model will give us useful inference of the faster oscillation in the presence of these slow modulations. We generated data with the offset, instead of being constant throughout a trial, to be a randomly generated square wave with a given duty cycle, and state duration generated from an exponential distribution with a minimum duration of 100 ms. Because the square wave is different in each trial, the overall PSTHs are flat. The spike train in Fig 8 have mean firing rates of 22Hz, a 20Hz oscillatory modulation with OCV = 0.27, with 200 trials used, and a duty cycle of 50% for the slow square wave. The resultant length was *L* = 0.25 ± 0.01. To analyze the data, we begin by inferring the slow time modulation using an AR(1) model as our latent model. We choose an AR(1) model, as we don’t expect very much structure in the slow latent state, and also because the time scale of the square waves relative to the duration of each trial ensures that any potential slow oscillatory structure would only be partially sampled, that considering additional structure in the AR is unnecessary. Although an AR(1) model is not the correct model describing these square waves, we are able to roughly infer the shape of the square waves, Fig 8 top.

We account for the slower fluctuation by modifying the CIF, Eq 3a to include the inferred slow fluctuation as a known signal *I*_*mn*_,

$$\begin{array}{c} p\left(y_{mn}=1|x_{mn},H_{n,l},\Theta \right)=\frac{e^{x_{mn}+\mu_{m}+f_{n}+\lambda_{l(m,n)}^{R}+I_{mn}}}{1+e^{x_{mn}+\mu_{m}+f_{n}+\lambda_{l(m,n)}^{R}+I_{mn}}}. \end{array}$$ (47)

After incorporating this known signal, we are able to infer the phases of the faster oscillation. Without including this step, the oscillatory AR(p) model would infer the slower fluctuation as in Fig 8 top, but inclusion of this known signal allows the faster oscillation to be inferred, Fig 8 bottom.

Neural data often contains more structure than the data analyst is aware of or suspects, and if on initial analysis with the standard LOST the analyst finds a much slower timescale latent state than expected, chances are the user has discovered a real but unknown feature of the data, and that the user may want to infer this slow latent state using an AR(1) as described in this section, and include this signal as a known signal in a second run of LOST to discover the higher-frequency modulation expected.

#### Mixture of modulated and non-modulated trials

**Fig 9:**
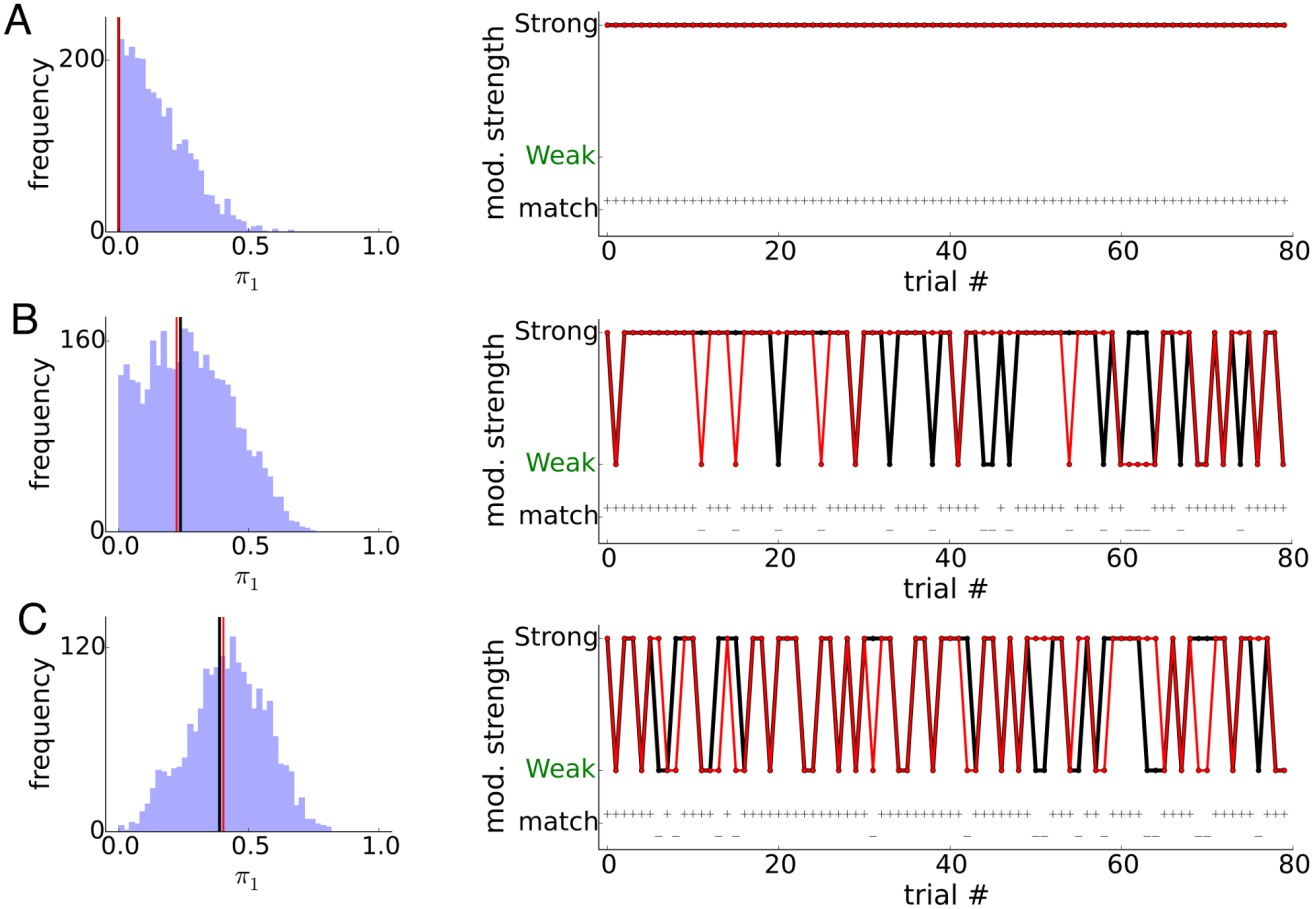
Mixture of modulated and non-modulated trials. A), B), C) 0%, 25% and 40% of the trials are weakly modulated. Left shows posterior distribution of *π*_1_ and the black line is the ground truth value, red line is the mode of the marginal posterior. Right shows inferred modulation state, weak or strong, for each of the 80 trials. Black dots are ground truth, red dots are inferred state. Below them are indicators (+−) of whether inferred matches ground truth.

Here we show an example where additional, discrete latent structures beside the oscillation, are present in the data. We motivate this extension to LOST as recent work has shown that brain states during sensory processing and behavior undergo discrete state transitions [39, 40], and because the authors have suspected (unpublished) that the motor cortical neurons analyzed in the Data Analysis section, also exhibit discrete transitions from trial to trial. Fig 9 shows results from 3 simulated spike trains, where 0% (Fig 9A), 25% (Fig 9B) or 40% (Fig 9C) of the of the trials are weakly modulated, and the rest strongly modulated. The mean firing rate for all 3 of the spiketrains are 36Hz, modulated with a 20Hz modulation with OCVs of 0.32. 80 trials were analyzed, each of duration 1.2 seconds.

The LOST model successfully infers the existence of 2 subsets of trials whose modulation strengths are very different in Fig 9B and 9C, and correctly infers that all trials are modulated by similar strengths in Fig 9A. To infer the modulation state, weak or strong, we compiled the posterior mean of the indicators 〈*Z*_*m*1_〉 for all trials *m*. Trials that are strongly modulated tend to have small values of 〈*Z*_*m*1_〉. The mixture coefficient ***π*** provides a straightforward threshold for 〈*Z*_*m*1_〉 for categorization into weak or strong trials. We choose the threshold to be $M\pi_{1}^{*}$, where $\pi_{1}^{*}$ is the posterior mode of *π*_1_. The trials whose 〈*Z*_*m*1_〉 are larger than the threshold are deemed weakly modulated trials. As can be seen in Fig 9B and 9C, the LOST model categorizes a significant fraction, > 80%, of trials correctly, and also infers the mixture weights close to the true values. We note that in inferring the value of *π*_1_, we did not take the mean as we do for most other parameters due to its bounded nature and the bias this confers when *π*_1_ is at or near 0, but rather take the mode of the posterior.

### Data analysis

**Fig 10:**
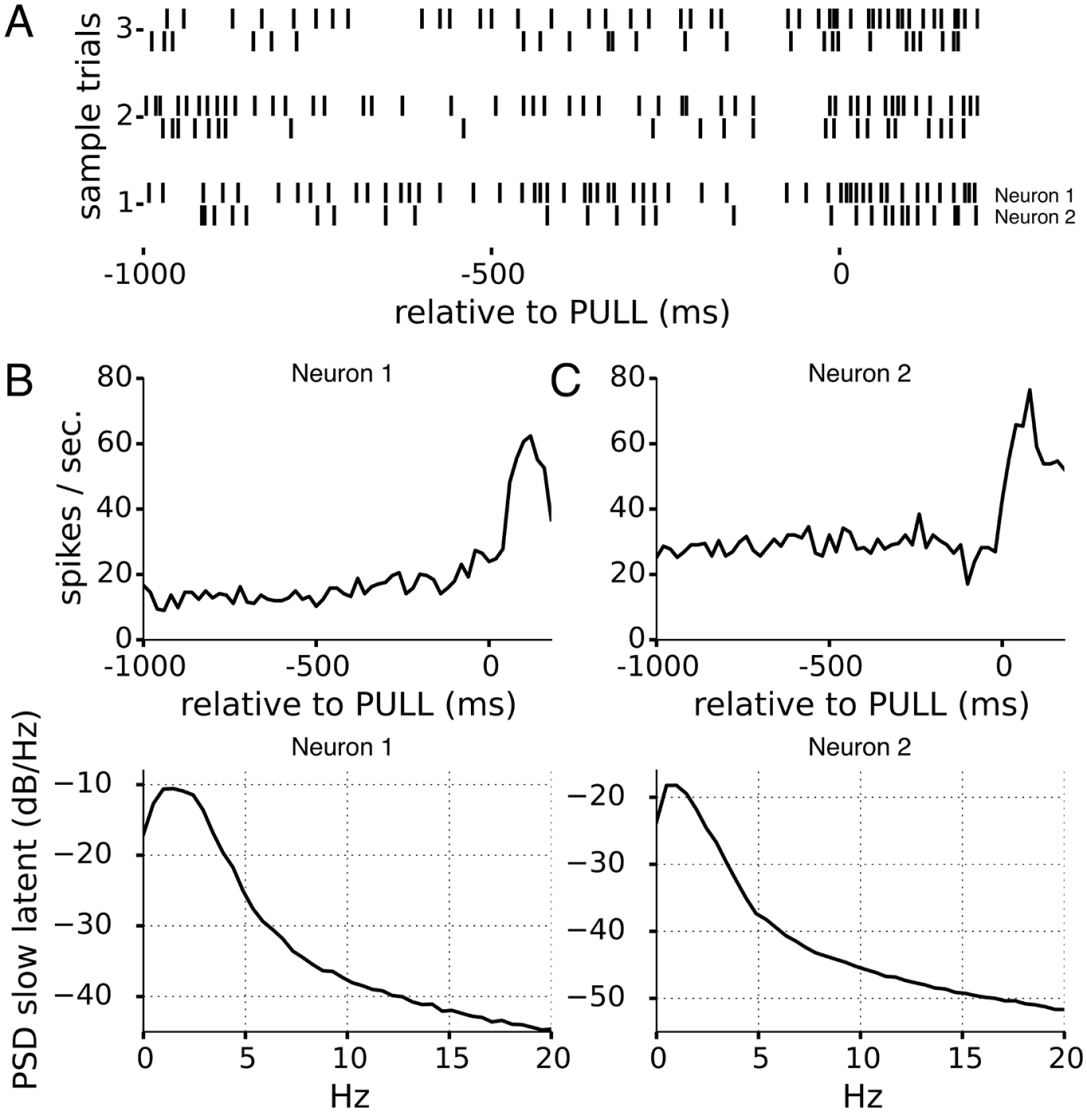
Two simultaneously-recorded motor cortical neurons: Slow fluctuations in firing rate for neuron 1 and 2. A) rasters from sample trials for the two neurons. There are noticeable holes in the rasters of the neuron 1, and neuron 2 also appears to have a similar slower fluctuation that is less noticeable. B, C) the PSTHs for the two neurons (top) show they both increase their activity around the time of PULL onset (0ms), and power spectral density of latent state show slow fluctuations on the order of 1-2 Hz. Correlation coefficient at 0 lag for the slow fluctuations inferred from neuron 1 and 2 are weakly correlated, with a correlation coefficient 0.15, *p* < 1 × 10^−3^, boot strap test.

**Fig 11:**
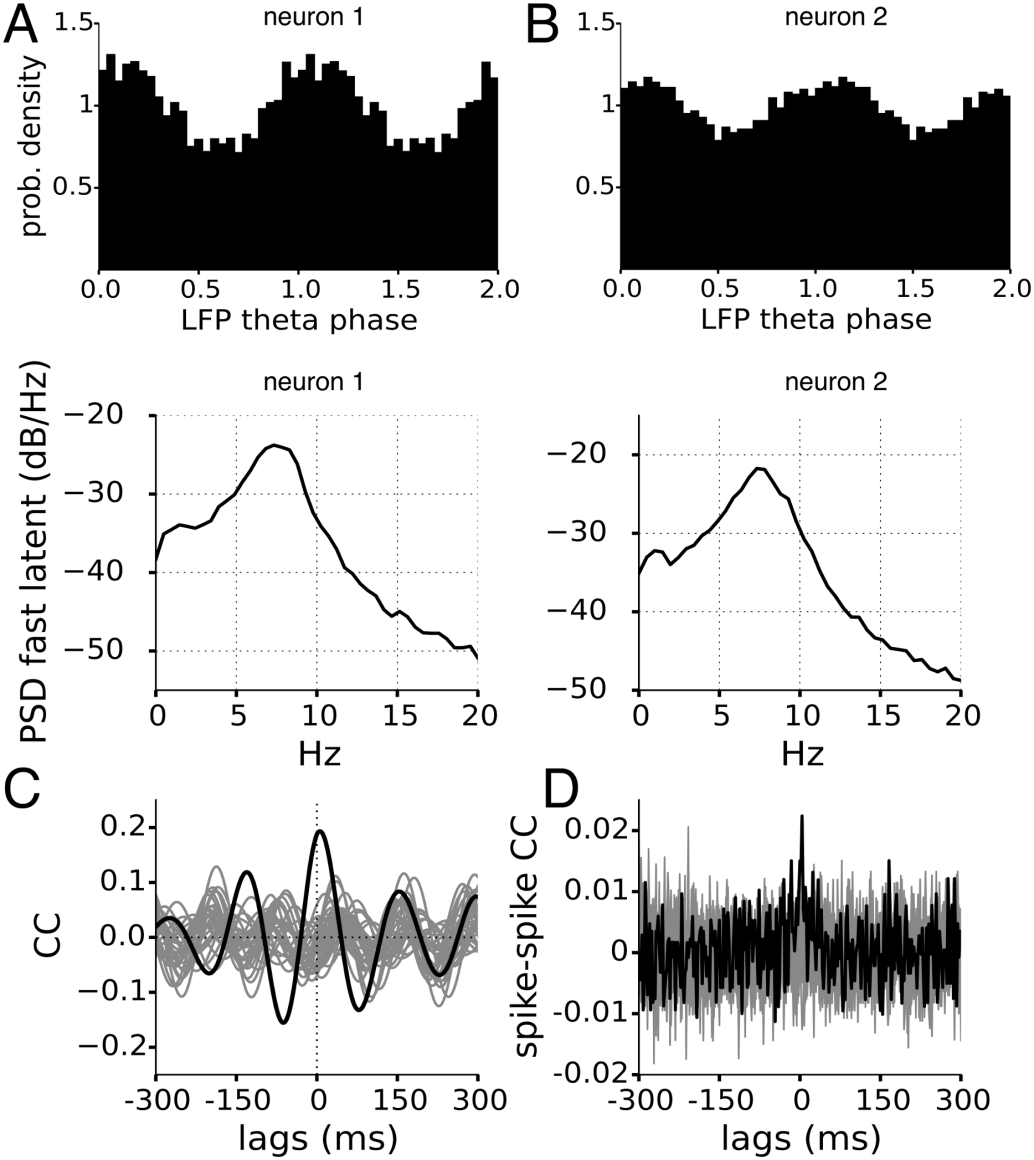
Two simultaneously-recorded motor cortical neurons: Simultaneous inference of theta oscillations using inferred slow fluctuation from Fig 10 as a known signal. A) SP histograms for neurons 1 and 2 with circular statistics R = 0.13 ± 0.01 and 0.08 ± 0.01. B) power spectral densities of inferred oscillation for the 2 neurons showing power in the theta range. C) The cross-correlation function of the inferred latent oscillations of the two neurons, grey traces from when trials shuffled. D) The spike-spike cross correlation function shows very little oscillatory structure.

We analyze neurons from M1 of rat performing a self-paced lever push-hold-pull task [13, 41] that have been found to be significantly modulated to the theta rhythm in the LFP. These 2 neurons were recorded on separate electrodes, with “neuron 1” in Figs 10 and 11 recorded and spike sorted from a tetrode on a siliconprobe, and “neuron 2” recorded using juxtacellular (cell-attached) recording, where the spike trains did not need to be sorted. Both neurons were located in layer 1/2, and “neuron 2” identified morphologically as an interneuron, while “neuron 1” is likely to be an interneuron based on spike shape. We identified trials by selecting lever hold periods lasting more than 1 second followed by a large-amplitude pull, and analyzed the 1.2 second period encompassing the hold-pull period.

#### Slow fluctuations

Fig 10A shows that the spike trains appear to have long periods of low firing rate, interspersed with periods of higher firing within a trial, creating noticeable holes in the spike train. Applying the LOST model using an AR(1) latent state first picks up a latent state that follows these slow fluctuations to the firing rate, but with the faster oscillatory modulation absent. Fig 10B and 10C bottom show the power spectrum for the fluctuation **I** we infer for each neuron, and find that both are slow, in the 1-2Hz range. Interestingly, this slow fluctuation is weakly correlated between the neurons, *p* < 6 × 10^−3^, bootstrap test.

#### Shared theta oscillations

Next, we account for the faster fluctuation by including the slow fluctuation **I** into the CIF as described in Eq 47. After incorporating this known signal, we are able to infer the phases, Fig 11 for both neurons. The power spectral density of the inferred latent oscillation are similar, with a peak around 7-8Hz, Fig 11A and 11B bottom. Igarashi *et al* [13] found significant modulation to the LFP theta of these neurons using SP histograms, but did not find direct evidence of simultaneous modulation of the neurons from the spike-spike cross-correlation function, Fig 11D. In contrast, the oscillatory cross-correlation function of the independently inferred oscillations shown in Fig 11C, clearly shows evidence of simultaneous modulation of the neurons to a shared oscillation.

#### Weakly and strongly modulated trials to LFP theta in a single neuron

**Fig 12:**
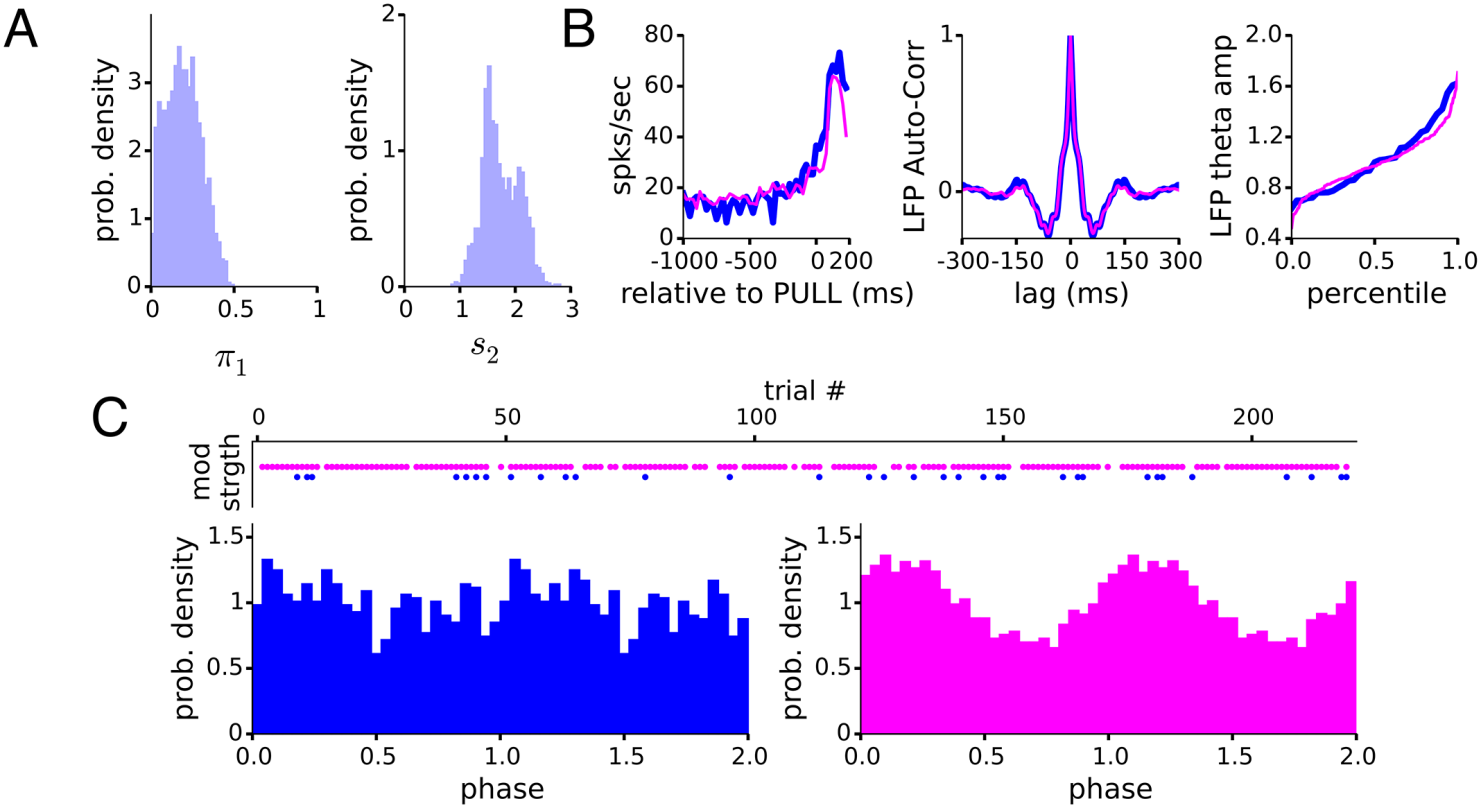
Motor cortical neuron (neuron 1, Figs 10 and 11) shows changing relationship to LFP theta oscillation in different trials. A) The posterior distributions of the mixture weight of the weakly modulated L trials, and relative modulation strength of the strongly modulated H trials. B) PSTH_L_ (blue) and PSTH_H_ (pink) overlap one another, showing no difference in average firing rate. C) LFP theta amplitudes calculated for all L and H trials on a per trial basis. Difference between cumulative distributions of per trial LFP theta amplitude not significant, *p* < 0.78, 2 sample KS-test. D) Top, the 33 (187) trials classified as weakly (strongly) modulated trials, blue (pink) dots, top. Bottom, the SP histogram calculated from L (H) trials, left (right). Inference of modulation strength using the spike train alone corresponds to the modulation strength to LFP theta. Circular statistics *R*_L_ = 0.06 ± 0.01 and *R*_H_ = 0.16 ± 0.01 are significantly different, bootstrap test *p* < 7 × 10^−4^.

In the previous section, we treated all trials of neuron 1 as equally modulated and inferred a theta modulation in its spike train. Using a simple model to fit often results in finding an oscillation, as we did for neuron 1, but it may be that there is further structure that can be discovered by using a more complicated parametric model. Here, we analyze neuron 1 using the mixture model, and find a significant difference in the strength of oscillatory modulation between trials. Following the procedure described for the mixture model, we find that about 85% of the trials (H, pink) have a much stronger modulation, Fig 12A right, than the remaining trials (L, blue), Fig 12C top.

We emphasize this classification was made using only the spike trains. The PSTHs constructed separately from L trials and H trials show no significant difference Fig 12B left, so the L and H trials being detected do not reflect a difference in temporal trial-locked firing rate, or in the baseline firing rate. Since the classification into L and H reflects a dichotomous change in the structure of oscillatory modulation, we expect to find a similar change in the relationship between the spike train and the LFP theta. It is possible that the L and H trials are indicative of absence or presence of the global LFP theta, that L trials show weak modulation because there is no modulating drive in those trials. The autocorrelation function of the high-pass filtered (above 1Hz) LFP constructed separately for the L trials and H trials, showed similar oscillatory structure, Fig 12B center. Further, the amplitude of the theta-band LFP in L trials and H trials also show no significant difference, Fig 12C, so global theta seems to be a reliable and perpetual presence. However, in Fig 12D, when we construct 2 separate LFP theta SP histograms, SP_L_ and SP_H_, we find that SP_L_ is significantly more uniform than SP_H_, with R_L_ = 0.06 ± 0.01 and R_H_ = 0.16 ± 0.01, bootstrap test *p* < 7 × 10^−4^. Thus it appears that this neuron intermittently participates in the perpetual theta rhythmicity, resulting in distinct subsets of trials, some being weakly modulated and others being strongly modulated.

## Discussion

We have defined the LOST model, together with accompanying posterior simulation technology, in order to detect the presence of oscillatory firing-rate modulation in a spike train, and infer its phase of oscillation in the presence of a variety of non-stationarities in firing rate that are present in experimental data. Previous methods have assessed the oscillatory content in spike trains by comparing the spiking to a known oscillatory signal like a band-passed LFP [42, 43], by detecting oscillation directly from the spike train [12, 18, 44–46], or by point process regression using the oscillatory signal as a covariate [47]. The LOST model not only detects oscillatory modulation from the spike train itself, but does so in the presence of both spiking noise and oscillatory irregularity, and also allows extensions to inferring additional latent structure, such as non-stationarities in the modulational strength across trials. In addition, LOST is able to separately account for modulational signals of different frequency band, which has proven to be vital in the analysis of real spike trains. Structural priors on the latent state dynamics together with explicit consideration of spiking noise and oscillatory irregularity allows LOST to uncover oscillatory structure even when the firing rate is low, the modulation weak or when the oscillation is irregular, compared to methods that directly regressed the spiking probability on the raw spiking history itself without an intermediary latent state [12, 18, 19]. LOST uses the intermediary latent state to addresses the irregularity characteristic of neural oscillations. Another approach to modeling the spectral features of time series using Gaussian processes has recently been developed by Wilson *et al* [48], where different classes of covariance kernels, the SE and SM, respectively, model non-oscillatory and oscillatory structures, analogous to the real and imaginary roots of the characteristic polynomials. It would be interesting to compare the two approaches in future work.

The increased sensitivity and flexibility in specifying latent structures, should allow LOST to be used in investigating the role of oscillations in cognitive functions in the cortex. Investigators are increasingly interested in characterizing the change in neural responses to time-varying stimulus or behavior [49, 50]. Theta and gamma oscillations in hippocampus change their coupling structure and prevalence during learning and memory acquisition [51, 52]. The inclusion of trial-specific structure independently of the LFP in the LOST model may also allow detection of increased recruitment of a given neuron into cell assemblies during periods when oscillations in the LFP are changing. Further, the variability of the timing and presence of oscillations in the LFP seen in many areas of the cortex and hippocampus [2, 13, 53], suggests oscillatory modulation in single neurons may likewise exhibit finer structure on a per-trial basis. Use of the LOST model in the analysis of such systems may reveal richer dynamics of recruitment into cell assemblies, and a better understanding of the role of single neurons in cognition.

## References

[1] Murthy VN, Fetz EE. Synchronization of neurons during local field potential oscillations in sensorimotor cortex of awake monkeys. Journal of neurophysiology. 1996 12;76(6):3968–82. Available from: http://www.ncbi.nlm.nih.gov/pubmed/8985893. 8985893 10.1152/jn.1996.76.6.3968

[2] Donoghue JP, Sanes JN, Hatsopoulos NG, Gaál G, Omlor W, Patino L, et al Neural Discharge and Local Field Potential Oscillations in Primate Motor Cortex During Voluntary Movements Neural Discharge and Local Field Potential Oscillations in Primate Motor Cortex During Voluntary Movements. Journal of neurophysiology. 1998;79:159–173. 9425187 10.1152/jn.1998.79.1.159

[3] Engel AK, Fries P, Singer W. Dynamic predictions: oscillations and synchrony in top-down processing. Nature reviews Neuroscience. 2001;2(10):704–716. 10.1038/35094565 11584308

[4] Siapas AG, Lubenov EV, Wilson Ma. Prefrontal phase locking to hippocampal theta oscillations. Neuron. 2005 4;46(1):141–51. Available from: http://www.ncbi.nlm.nih.gov/pubmed/15820700. 10.1016/j.neuron.2005.02.028 15820700

[5] Denker M, Roux S, Timme M, Riehle A, Grün S. Phase synchronization between LFP and spiking activity in motor cortex during movement preparation. Neurocomputing. 2007;70(10-12):2096–2101. 10.1016/j.neucom.2006.10.088

[6] Gregoriou GG, Gotts SJ, Zhou H, Desimone R. High-frequency, long-range coupling between prefrontal and visual cortex during attention. Science. 2009;324(5931):1207–1210. 10.1126/science.1171402 19478185 PMC2849291

[7] Fries P, Womelsdorf T, Oostenveld R, Desimone R. The effects of visual stimulation and selective visual attention on rhythmic neuronal synchronization in macaque area V4. The Journal of neuroscience: the official journal of the Society for Neuroscience. 2008;28(18):4823–4835. 10.1523/JNEUROSCI.4499-07.2008 18448659 PMC3844818

[8] Siegel M, Warden MR, Miller EK. Phase-dependent neuronal coding of objects in short-term memory. Proceedings of the National Academy of Sciences. 2009 12;106(50):21341–6. Available from: http://www.pubmedcentral.nih.gov/articlerender.fcgi?artid=2779828{&}tool=pmcentrez{&}rendertype=abstract. 10.1073/pnas.0908193106 PMC2779828 19926847

[9] Benchenane K, Peyrache A, Khamassi M, Tierney PL, Gioanni Y, Battaglia FP, et al Coherent theta oscillations and reorganization of spike timing in the hippocampal- prefrontal network upon learning. Neuron. 2010 6;66(6):921–36. Available from: http://www.ncbi.nlm.nih.gov/pubmed/20620877. 10.1016/j.neuron.2010.05.013 20620877

[10] Ching S, Cimenser A, Purdon PL, Brown EN, Kopell NJ. Thalamocortical model for a propofol-induced *α*-rhythm associated with loss of consciousness. Proceedings of the National Academy of Sciences. 2010;107(52):22665–22670. Available from: http://www.pnas.org/cgi/doi/10.1073/pnas.1017069108. 10.1073/pnas.1017069108 PMC3012501 21149695

[11] Chalk M, Herrero JL, Gieselmann Ma, Delicato LS, Gotthardt S, Thiele A. Attention reduces stimulus-driven gamma frequency oscillations and spike field coherence in V1. Neuron. 2010 4;66(1):114–25. Available from: http://www.pubmedcentral.nih.gov/articlerender.fcgi?artid=2923752{&}tool=pmcentrez{&}rendertype=abstract. 10.1016/j.neuron.2010.03.013 20399733 PMC2923752

[12] Sarma SV, Eden UT, Cheng ML, Williams ZM, Hu R, Eskandar E, et al Using point process models to compare neural spiking activity in the subthalamic nucleus of Parkinson’s patients and a healthy primate. IEEE transactions on bio-medical engineering. 2010 6;57(6):1297–305. Available from: http://www.ncbi.nlm.nih.gov/pubmed/20172804. 10.1109/TBME.2009.2039213 20172804 PMC3822781

[13] Igarashi J, Isomura Y, Arai K, Harukuni R, Fukai T. Oscillation Code for Neuronal Coordination during Motor Behavior. Journal of Neuroscience. 2013 11;33(47):18515–18530. Available from: http://www.jneurosci.org/cgi/doi/10.1523/JNEUROSCI.2126-13.2013. 24259574 10.1523/JNEUROSCI.2126-13.2013 PMC6618805

[14] Mukamel EA, Pirondini E, Babadi B, Wong KFK, Pierce ET, Harrell PG, et al A transition in brain state during propofol-induced unconsciousness. Journal of Neuroscience. 2014;34(3):839–845. Available from: http://www.jneurosci.org/cgi/doi/10.1523/JNEUROSCI.5813-12.2014. 24431442 10.1523/JNEUROSCI.5813-12.2014 PMC3891963

[15] Fries P. A mechanism for cognitive dynamics: neuronal communication through neuronal coherence. Trends in cognitive sciences. 2005 10;9(10):474–80. Available from: http://www.ncbi.nlm.nih.gov/pubmed/16150631. 10.1016/j.tics.2005.08.011 16150631

[16] Fell J, Axmacher N. The role of phase synchronization in memory processes. Nature reviews Neuroscience. 2011 2;12(2):105–18. Available from: http://www.ncbi.nlm.nih.gov/pubmed/21248789. 10.1038/nrn2979 21248789

[17] Denker M, Roux S, Lindén H, Diesmann M, Riehle A, Grün S. The local field potential reflects surplus spike synchrony. Cerebral Cortex. 2011;21(12):2681–2695. 10.1093/cercor/bhr040 21508303 PMC3209854

[18] Eden UT, Gale JT, Amirnovin R, Eskandar EN. Characterizing the spiking dynamics of subthalamic nucleus neurons in Parkinson’s disease using generalized linear models. Frontiers in integrative neuroscience. 2012 1;6(June):28 Available from: http://www.pubmedcentral.nih.gov/articlerender.fcgi?artid=3379030{&}tool=pmcentrez{&}rendertype=abstract. 10.3389/fnint.2012.00028 22723771 PMC3379030

[19] Deng X, Eskandar EN, Eden UT. A point process approach to identifying and tracking transitions in neural spiking dynamics in the subthalamic nucleus of Parkinson’s patients. Chaos. 2013 12;23(4):046102 Available from: http://www.pubmedcentral.nih.gov/articlerender.fcgi?artid=3808419{&}tool=pmcentrez{&}rendertype=abstract. 10.1063/1.4818546 24387581 PMC3808419

[20] Truccolo W, Eden UT, Fellows MR, Donoghue JP, Brown EN. A point process framework for relating neural spiking activity to spiking history, neural ensemble, and extrinsic covariate effects. Journal of neurophysiology. 2005;93(September 2004):1074–1089. 15356183 10.1152/jn.00697.2004

[21] Kass RE, Eden UT, Brown EN. Analysis of Neural Data. Springer; 2014.

[22] Smith AC, Brown EN. Estimating a state-space model from point process observations. Neural computation. 2003 5;15(5):965–91. Available from: http://www.ncbi.nlm.nih.gov/pubmed/12803953. 10.1162/089976603765202622 12803953

[23] Kitagawa G. Introduction to Time Series Modeling. Chapman & Hall / CRC Monographs on Statistics & Applied Probability; 2010.

[24] Huerta G, West M. Priors and component structures in autoregressive time series models. Journal of the Royal Statistics Society Series B. 1999;61(4):881–899. 10.1111/1467-9868.00208

[25] Polson NG, Scott JG, Windle J. Bayesian Inference for Logistic Models Using Pólya-Gamma Latent Variables. Journal of the American Statistical Association. 2013;108(504):1339–1349. Available from: http://www.tandfonline.com/doi/abs/10.1080/01621459.2013.829001.

[26] Allcroft DJ, Glasbey CA. A spectral estimator of ARMA parameters from thresholded data. Statistics and Computing. 2002;12:369–376. 10.1023/A:1020796314300

[27] Kass RE, Ventura V. A Spike-Train Probability Model. Neural Computation. 2001;1720:1713–1720. 10.1162/08997660152469314 11506667

[28] Gelman A, Carlin JB, Stern HS, Rubin DB. Bayesian Data Analysis. Chapman & Hall; 1995.

[29] Tanner MA, Wong WH. The Calculation of Posterior Distributions by Data Augmentation. Journal of the American Statistical Association. 1987 6;82(398):528–540. Available from: http://www.tandfonline.com/doi/abs/10.1080/01621459.1987.10478458. 10.2307/2289463

[30] Frühwirth-Schnatter S. Data Augmentation and Dynamic Linear Models. Journal of Time Series Analysis. 1994;15(2):183–202. 10.1111/j.1467-9892.1994.tb00184.x

[31] Penttonen M, Kamondi A, Acsady L, Buzsaki G. Gamma frequency oscillation in the hippocampus of the rat: intracellular analysis in vivo. European Journal of Neuroscience. 1998 2;10(2):718–728. Available from: http://doi.wiley.com/10.1046/j.1460-9568.1998.00096.x. 9749733 10.1046/j.1460-9568.1998.00096.x

[32] Burgess N, O’Keefe J. Models of place and grid cell firing and theta rhythmicity. Current opinion in neurobiology. 2011 10;21(5):734–44. Available from: http://www.pubmedcentral.nih.gov/articlerender.fcgi?artid=3223517{&}tool=pmcentrez{&}rendertype=abstract. 10.1016/j.conb.2011.07.002 21820895 PMC3223517

[33] Polasek W, Jin S. Gibbs Sampling in AR Models with Random Walk Priors In: From Data to Knowledge: Theoretical and practical aspects of classification, data analysis, and knowledge organization. Springer; 1996 p. 86–93.

[34] Funkhouser HG. A Short Account of the History of Symmetric Functions of Roots of Equations. The American Mathematical Monthly. 1930;37(7):357–365. 10.2307/2299273

[35] Buesing L, Macke JH, Sahani M. Learning stable, regularised latent models of neural population dynamics. Network: Computation in Neural Systems. 2012;23(1-2):24–47. 10.3109/0954898X.2012.677095 22663075

[36] Sherman BYJ, Morrison WJ. Adjustment of an Inverse Matrix Corresponding to a Change in One Element of a Given Matrix. The Annals of Mathematical Statistics. 1950;21(1):124–127. 10.1214/aoms/1177729893

[37] Brown EN, Nguyen DP, Frank LM, Wilson Ma, Solo V. An analysis of neural receptive field plasticity by point process adaptive filtering. Proceedings of the National Academy of Sciences. 2001;98(21):12261–12266. 10.1073/pnas.201409398 PMC59830 11593043

[38] Okamoto H, Isomura Y, Takada M, Fukai T. Temporal integration by stochastic recurrent network dynamics with bimodal neurons. Journal of neurophysiology. 2007;97(6):3859–3867. 10.1152/jn.01100.2006 17392417

[39] Jones LM, Fontanini A, Sadacca BF, Miller P, Katz DB. Natural stimuli evoke dynamic sequences of states in sensory cortical ensembles. Proceedings of the National Academy of Sciences. 2007;104(47):18772–18777. Available from: http://www.pubmedcentral.nih.gov/articlerender.fcgi?artid=2141852{&}tool=pmcentrez{&}rendertype=abstract. 10.1073/pnas.0705546104 PMC2141852 18000059

[40] Ulrich KR, Carlson DE, Lian W, Borg JS, Dzirasa K, Carin L. Analysis of Brain States from Multi-Region LFP Time-Series In: Ghahramani Z, Welling M, Cortes C, Lawrence ND, Weinberger KQ, editors. Advances in Neural Information Processing Systems 27. Curran Associates, Inc.; 2014 p. 2483–2491. Available from: http://papers.nips.cc/paper/5624-analysis-of-brain-states-from-multi-region-lfp-time-series.pdf.

[41] Isomura Y, Harukuni R, Takekawa T, Aizawa H, Fukai T. Microcircuitry coordination of cortical motor information in self-initiation of voluntary movements. Nature neuroscience. 2009 12;12(12):1586–93. Available from: http://www.ncbi.nlm.nih.gov/pubmed/19898469. 10.1038/nn.2431 19898469

[42] Fries P, Roelfsema PR, Engel AK, König P, Singer W. Synchronization of oscillatory responses in visual cortex correlates with perception in interocular rivalry. Proceedings of the National Academy of Sciences. 1997;94(November):12699–12704. 10.1073/pnas.94.23.12699 PMC25091 9356513

[43] Lepage KQ, Gregoriou GG, Kramer MA, Aoi M, Gotts SJ, Eden UT, et al A procedure for testing across-condition rhythmic spike-field association change. Journal of neuroscience methods. 2013 2;213(1):43–62. Available from: http://www.ncbi.nlm.nih.gov/pubmed/23164959. 10.1016/j.jneumeth.2012.10.010 23164959 PMC3800189

[44] Mureşan RC, Jurjuţ OF, Moca VV, Singer W, Nikolić D. The Oscillation Score: An Efficient Method for Estimating Oscillation Strength in Neuronal Activity. Journal of neurophysiology. 2008;p. 1333–1353. 18160427 10.1152/jn.00772.2007

[45] Climer JR, Newman EL, Hasselmo ME. Phase coding by grid cells in unconstrained environments: two-dimensional phase precession. The European journal of neuroscience. 2013 8;38(4):2526–41. Available from: http://www.pubmedcentral.nih.gov/articlerender.fcgi?artid=3912569{&}tool=pmcentrez{&}rendertype=abstract. 10.1111/ejn.12256 23718553 PMC3912569

[46] Matzner A, Bar-Gad I. Quantifying Spike Train Oscillations: Biases, Distortions and Solutions. PLOS Computational Biology. 2015;11(4):e1004252 Available from: http://dx.plos.org/10.1371/journal.pcbi.1004252. 25909328 10.1371/journal.pcbi.1004252 PMC4409360

[47] Zhou P, Burton SD, Snyder AC, Smith MA, Urban NN, Kass RE. Establishing a Statistical Link between Network Oscillations and Neural Synchrony. PLoS Computational Biology. 2015;11(10):1–25. 10.1371/journal.pcbi.1004549 PMC4605746 26465621

[48] Wilson AG, Adams RP. Gaussian Process Covariance Kernels for Pattern Discovery and Extrapolation. In: Proceedings of the 30th International Conference on Machine Learning. vol. 28; 2013. p. 15. Available from: http://arxiv.org/abs/1302.4245.

[49] Czanner G, Eden UT, Wirth S, Yanike M, Suzuki Wa, Brown EN. Analysis of between-trial and within-trial neural spiking dynamics. Journal of neurophysiology. 2008;99(5):2672–2693. 10.1152/jn.00343.2007 18216233 PMC2430469

[50] Mandelblat-Cerf Y, Paz R, Vaadia E. Trial-to-trial variability of single cells in motor cortices is dynamically modified during visuomotor adaptation. The Journal of neuroscience. 2009 12;29(48):15053–62. Available from: http://www.ncbi.nlm.nih.gov/pubmed/19955356. 10.1523/JNEUROSCI.3011-09.2009 19955356 PMC6665974

[51] Tort ABL, Komorowski RW, Manns JR, Kopell NJ, Eichenbaum H. Theta-gamma coupling increases during the learning of item-context associations. Proceedings of the National Academy of Sciences. 2009;106(49):20942–20947. 10.1073/pnas.0911331106 PMC2791641 19934062

[52] van Vugt MK, Schulze-Bonhage A, Litt B, Brandt A, Kahana MJ. Hippocampal gamma oscillations increase with memory load. The Journal of Neuroscience. 2010;30(7):2694–2699. 10.1523/JNEUROSCI.0567-09.2010 20164353 PMC2835496

[53] Yartsev MM, Witter MP, Ulanovsky N. Grid cells without theta oscillations in the entorhinal cortex of bats. Nature. 2011 11;479(7371):103–7. Available from: http://www.ncbi.nlm.nih.gov/pubmed/22051680. 10.1038/nature10583 22051680

